# Hesitant Fuzzy Subalgebras, Ideals and Congruences on Autometrized Algebras

**DOI:** 10.12688/f1000research.161430.2

**Published:** 2025-09-09

**Authors:** Gebrie Yeshiwas Tilahun

**Affiliations:** 1 Department of Mathematics, Assosa University, Asosa, Benishangul-Gumuz, Ethiopia

**Keywords:** autometrized algebra, hesitant fuzzy subalgebra, hesitant fuzzy ideal, hesitant fuzzy congruence

## Abstract

This paper introduces the study of hesitant fuzzy subalgebras of autometrized algebras, obtains some of their properties, and gives some examples. Next, we introduce the concept of the hesitant fuzzy ideal and examine some of its properties. Finally, we introduce a hesitant fuzzy congruence on autometrized algebras and discuss some of its properties. We also introduce the characteristic equations and level subsets of hesitant fuzzy sets and relations on autometrized algebras.

## 1. Introduction

In his work, ^1^ introduced the concept of autometrized algebras which includes Boolean algebras, ^2^^,^^3^ Brouwerian algebras, ^4^ Newman algebras, ^5^ autometrized lattices, ^4^ and commutative lattice-ordered groups (or l-groups). ^6^ The study of ideals and congruences in autometrized algebras was conducted by Ref. 7. Further advancements in the theory of autometrized algebras were made by Refs. 7– 22 developed the theory of subalgebras, ideals, and congruences of autometrized algebras. Moreover, Ref. 23 introduced the homomorphism, isomorphism, and correspondence theorems of autometrized algebra by using congruency.

The concept of fuzzy sets was introduced by Refs. 24– 26 introduced the concept of hesitant fuzzy sets. Several researches were conducted on the generalizations of the notion of hesitant fuzzy sets and its applications such as Refs. 27– 34. Recent studies have introduced various concepts, including to fuzzy soft group decision-making using weighted average ratings, ^35^ parameterized intuitionistic fuzzy soft multiset and group decision-making, ^36^ IFP-intuitionistic multi-fuzzy N-soft set and its induced IFP-hesitant N-soft set in decision-making, ^37^ weighted hesitant bipolar-valued fuzzy soft set in decision-making ^38^ and its corresponding IFP-hesitant N-soft set. ^39^ These studies demonstrated that hesitant fuzzy sets are increasingly applied in multi-criteria decision-making, where decision-makers may be hesitant about different membership values for specific criteria. Additionally, they contributed to the creation of an efficient model for evaluating water quality. ^40^ introduced fuzzy set theory applied on autometrized algebras.

Classical set theory and traditional fuzzy sets assign a single membership degree to each element. However, these approaches often fall short in addressing real-world problems that exhibit ambiguity, hesitation, or conflicting preferences. In such scenarios, classical set theory and even basic fuzzy set theory may not fully capture the complexities of decision-makers’ uncertainty. To address these limitations, the concept of hesitant fuzzy sets was introduced as a generalization of fuzzy sets. The key difference between fuzzy sets and hesitant fuzzy sets is that hesitant fuzzy set theory enhances fuzzy logic by allowing multiple membership degrees for an element within a set, thereby reflecting the hesitation among various possible values. Moreover, previous studies did not investigate hesitant fuzzy subalgebra, hesitant fuzzy ideal, and hesitant fuzzy congruence of autometrized algebra. Therefore, our motivation is to address these gaps. This paper introduces the concept of hesitant fuzzy subalgebras, hesitant fuzzy ideals, and hesitant fuzzy congruence relations on autometrized algebras.

The paper will be organized as follows: Section 2 will provide definitions and key terms. Section 3 will introduce the concept of hesitant fuzzy subalgebras of autometrized algebras. In Section 4, we will discuss hesitant fuzzy ideals of autometrized algebras. Section 5 will focus on hesitant fuzzy congruences on autometrized algebras. Finally, Section 6 will conclude the paper.

In this paper, Γ denotes an autometrized algebra (Γ, +, 0, ≼, ⋆).

## 2. Preliminaries

This section examines essential concepts, definitions, and theorems important in other sections.

Definition 2.1 ^1^ *A system* Γ=(Γ,+,0,≼,⋆) *is called an autometrized algebra if*

(i)
(Γ,+,0) *is a commutative monoid.*
(ii)
(Γ,≼) *is a partial ordered set, and* ≼ *is translation invariant, that is,* ∀α,β,γ∈Γ;α≼β⇒α+γ≼β+γ .
(iii)
⋆:Γ×Γ→Γ *is autometric on* Γ , *that is,* ⋆ *satisfies metric operation axioms:*
( *M*1) ∀α,β∈Γ;α⋆β≽0 *and*, α⋆β=0⇔α=β ,
( *M*2) ∀α,β∈Γ;α⋆β=β⋆α ,
( *M*3) ∀α,β,γ∈Γ ; α⋆γ≼α⋆β+β⋆γ .

Definition 2.2 ^7^ Γ *is called normal if and only if*

(i)
α≼α⋆0∀α∈Γ *.*
(ii)
(α+γ)⋆(β+δ)≼(α⋆β)+(γ⋆δ)∀α,β,γ,δ∈Γ *.*
(iii)
(α⋆γ)⋆(β⋆δ)≼(α⋆β)+(γ⋆δ)∀α,β,γ,δ∈Γ *.*
(iv) *For any* α *and* β *in* Γ *,* α≼β⇒∃γ≽0 *such that* α+γ=β *.*

Definition 2.3 ^19^ *Let* ϒ⊆Γ *. Then* ϒ *is said to be a subalgebra of* Γ *if;*

(i)
(ϒ,+,0) *is a commutative monoid.*
(ii)
(ϒ,≼) *is a subposet of Γ , and* ≼ *is translation invariant, that is,* α≼β⇒α+γ≼β+γ *for any* α,β,γ∈ϒ *.*
(iii)
⋆|ϒ:ϒ×ϒ→ϒ *is metric.*

Definition 2.4 ^19^ *A nonempty subset* I *of* Γ *is called an ideal if and only if*

(i)
α,β∈I *imply* α+β∈I *.*
(ii)
α∈I,β∈Γ *and* β⋆0≼α⋆0 *imply* β∈I *.*

Definition 2.5 ^7^ *An equivalence relation* Ψ *on* Γ *is called a congruence relation if and only if*

(i)
(α,β),(γ,δ)∈Ψ⇒(α+γ,β+δ)∈Ψ∀α,β,γ,δ∈Γ ,
(ii)
(α,β),(γ,δ)∈Ψ⇒(α⋆γ,β⋆δ)∈Ψ∀α,β,γ,δ∈Γ ,
(iii)
(α,β)∈Ψandγ⋆δ≼α⋆β⇒(γ,δ)∈Ψ∀α,β,γ,δ∈Γ .

Definition 2.6 ^24^ *Let* Γ *be a nonempty set, a fuzzy subset* χ *of* Γ *is a mapping* χ:Γ→[0,1] *.*

Definition 2.7 ^24^ *Let* Γ *be a nonempty set and* χ *be a fuzzy subset of* Γ *, for* ε∈[0,1] *, the set* $\chi_{\varepsilon }=\left\{\alpha \in \Gamma |\chi \left(\alpha \right)\geq \varepsilon \right\}$ *is called a level subset of* χ *.*

Definition 2.8 ^26^ *Let* Γ *be a reference set. A hesitant fuzzy set on* Γ *is a mapping* χ:Γ→P([0,1]) *, where* P([0,1]) *means the power set of* [0,1] *.*

Definition 2.9 ^26^ *Let* Γ *be a reference set. If* H⊆Γ *, the characteristic hesitant fuzzy set* $\chi_{H}$ *on* Γ *is a function of* Γ *into* P([0,1]) *defined as for all* α∈Γ *:*

$$\chi_{H}\left(\alpha \right)=\left\{ \begin{array}{ll} \left[0,\;1\right], & \text{if}\;\alpha \in H.\\ \emptyset , & \text{otherwise}. \end{array}\right.$$

We will denote the set of all hesitant fuzzy sets on Γ as *HS* ( Γ ).

Definition 2.10 ^41^ *Let* $\chi_{1},\chi_{2}\in \mathrm{HS}\left(\Gamma \right).$ *Then,*

(i) *We say that* $\chi_{1}$ *is a subset of* $\chi_{2}$ *, denoted by* $\chi_{1}\subseteq \chi_{2}$ *, if* $\chi_{1}\left(a\right)\subseteq \chi_{2}\left(a\right)$ *, for* a∈Γ .
(ii) *We say that* $\chi_{1}$ *is equal to* $\chi_{2}$ *, denoted by* $\chi_{1}=\chi_{2}$ *, if* $\chi_{1}\subseteq \chi_{2}$ *and* $\chi_{2}\subseteq \chi_{1}.$

Definition 2.11 ^42^ *Let* $\chi_{1},\chi_{2}\in \mathrm{HS}\left(\Gamma \right)$ and let ${\left(\chi_{j}\right)}_{j\in J}\subseteq \mathrm{HS}\left(\Gamma \right).$ *Then,*

(i) *the intersection of* $\chi_{1}$ *and* $\chi_{2}$ *, denoted by* $\chi_{1}\cap \chi_{2}$ *, is a hesitant fuzzy set on* Γ *defined as for each* a∈Γ , $\left(\chi_{1}\cap \chi_{2}\right)\left(a\right)=\chi_{1}\left(a\right)\cap \chi_{2}\left(a\right)$ .
(ii) *the intersection of* ${\left(\chi_{j}\right)}_{j\in J}$ *, denoted by* $\cap_{j\in J}\chi_{j}$ *, is a hesitant fuzzy set on* Γ *defined as for each* a∈Γ , $\left(\cap_{j\in J}\chi_{j}\right)\left(a\right)=\cap_{j\in J}\chi_{j}\left(a\right)$ .
(iii) *the union of* $\chi_{1}$ and $\chi_{2}$ *, denoted by* $\chi_{1}\cup \chi_{2}$ *, is a hesitant fuzzy set on* Γ *defined as for each* a∈Γ , $\left(\chi_{1}\cup \chi_{2}\right)\left(a\right)=\chi_{1}\left(a\right)\cup \chi_{2}\left(a\right)$ .
(iv) *the union of* ${\left(\chi_{j}\right)}_{j\in J}$ *, denoted by* $\cup_{j\in J}\chi_{j}$ *, is a hesitant fuzzy set on* Γ *defined as for each* a∈Γ , $\left(\cup_{j\in J}\chi_{j}\right)\left(a\right)=\cup_{j\in J}\chi_{j}\left(a\right)$ .

Definition 2.12 ^26^ *Let* χ *be a hesitant fuzzy set on a nonempty set* Γ *. Then,* $\bar{\chi }\left(\alpha \right)=\left[0,\;1\right]\backslash \chi \left(\alpha \right)$ *for all* α∈Γ *which is said to be the complement of* χ *on* Γ *.*

## 3. Hesitant Fuzzy Subalgebra of Autometrized Algebra

In this section, we will introduce the hesitant fuzzy subalgebras of autometrized algebras and explore several fundamental properties related to these subalgebras.

Definition 3.1 *A hesitant fuzzy subset* χ *of* Γ *is called a hesitant fuzzy subalgebra of* Γ *if for all* α,β∈Γ *;*

(i)
χ(α+β)⊇χ(α)∩χ(β) .
(ii)
χ(α⋆β)⊇χ(α)∩χ(β) .

Example 3.2 *Let* Γ={0,α,β,γ} *with* 0≼α,β≼γ *and elements* α,β *are incomparable. Define* ⋆ *and* + *by the following tables.*

**Table:** 

⋆	0	α	β	γ
0	0	α	β	γ
α	α	0	γ	β
β	β	γ	0	α
γ	γ	β	α	0

**Table:** 

+	0	α	β	γ
0	0	α	β	γ
α	α	α	γ	γ
β	β	γ	β	γ
γ	γ	γ	γ	γ

*Then,* Γ *is an autometrized algebra. Define a hesitant fuzzy subset* χ:Γ→P([0,1]) *of* Γ *by:* χ(0)={0.3,0.5} *and* χ(α)=χ(β)=χ(γ)={0.5} *. Now, check the two closure properties,*

(i)
$$\begin{array}{l} \chi \left(0+\alpha \right)=\chi \left(\alpha \right)=\left\{0.5\right\}⊇\chi \left(0\right)\cap \chi \left(\alpha \right)=\left\{0.5\right\}\\ \chi \left(0+\beta \right)=\chi \left(\beta \right)=\left\{0.5\right\}⊇\chi \left(0\right)\cap \chi \left(\beta \right)=\left\{0.5\right\}\\ \chi \left(0+\gamma \right)=\chi \left(\gamma \right)=\left\{0.5\right\}⊇\chi \left(0\right)\cap \chi \left(\gamma \right)=\left\{0.5\right\}\\ \chi \left(\alpha +\alpha \right)=\chi \left(\alpha \right)=\left\{0.5\right\}⊇\chi \left(\alpha \right)\cap \chi \left(\alpha \right)=\left\{0.5\right\}\\ \chi \left(\alpha +\beta \right)=\chi \left(\gamma \right)=\left\{0.5\right\}⊇\chi \left(\alpha \right)\cap \chi \left(\beta \right)=\left\{0.5\right\}\\ \chi \left(\alpha +\gamma \right)=\chi \left(\gamma \right)=\left\{0.5\right\}⊇\chi \left(\alpha \right)\cap \chi \left(\gamma \right)=\left\{0.5\right\}\\ \chi \left(\beta +\beta \right)=\chi \left(\beta \right)=\left\{0.5\right\}⊇\chi \left(\beta \right)\cap \chi \left(\beta \right)=\left\{0.5\right\}\\ \chi \left(\beta +\gamma \right)=\chi \left(\gamma \right)=\left\{0.5\right\}⊇\chi \left(\beta \right)\cap \chi \left(\gamma \right)=\left\{0.5\right\}\\ \chi \left(\gamma +\gamma \right)=\chi \left(\gamma \right)=\left\{0.5\right\}⊇\chi \left(\gamma \right)\cap \chi \left(\gamma \right)=\left\{0.5\right\} \end{array}$$
(ii)
$$\begin{array}{l} \chi \left(0⋆\alpha \right)=\chi \left(\alpha \right)=\left\{0.5\right\}⊇\chi \left(0\right)\cap \chi \left(\alpha \right)=\left\{0.5\right\}\\ \chi \left(0⋆\beta \right)=\chi \left(\beta \right)=\left\{0.5\right\}⊇\chi \left(0\right)\cap \chi \left(\beta \right)=\left\{0.5\right\}\\ \chi \left(0⋆\gamma \right)=\chi \left(\gamma \right)=\left\{0.5\right\}⊇\chi \left(0\right)\cap \chi \left(\gamma \right)=\left\{0.5\right\}\\ \chi \left(\alpha ⋆\alpha \right)=\chi \left(0\right)=\left\{0.3,0.5\right\}⊇\chi \left(\alpha \right)\cap \chi \left(\alpha \right)=\left\{0.5\right\}\\ \chi \left(\alpha ⋆\beta \right)=\chi \left(\gamma \right)=\left\{0.5\right\}⊇\chi \left(\alpha \right)\cap \chi \left(\beta \right)=\left\{0.5\right\}\\ \chi \left(\alpha ⋆\gamma \right)=\chi \left(\beta \right)=\left\{0.5\right\}⊇\chi \left(\alpha \right)\cap \chi \left(\gamma \right)=\left\{0.5\right\}\\ \chi \left(\beta ⋆\beta \right)=\chi \left(0\right)=\left\{0.3,0.5\right\}⊇\chi \left(\beta \right)\cap \chi \left(\beta \right)=\left\{0.5\right\}\\ \chi \left(\beta ⋆\gamma \right)=\chi \left(\alpha \right)=\left\{0.5\right\}⊇\chi \left(\beta \right)\cap \chi \left(\gamma \right)=\left\{0.5\right\}\\ \chi \left(\gamma ⋆\gamma \right)=\chi \left(0\right)=\left\{0.3,0.5\right\}⊇\chi \left(\gamma \right)\cap \chi \left(\gamma \right)=\left\{0.5\right\} \end{array}$$

*Therefore,* χ *is hesitant fuzzy subalgebra of* Γ *.*

Theorem 3.3 *If* χ *is a hesitant fuzzy subalgebra of* Γ *, then for* α∈Γ *;* χ(0)⊇χ(α) *.*

Proof.
Assume that χ is a hesitant fuzzy subalgebra of Γ . Then, χ(α⋆α)⊇χ(α)∩χ(α) . This implies that χ(0)⊇χ(α)∩χ(α) . Therefore, χ(0)⊇χ(α) .

Theorem 3.4 *Let* H *is a nonempty subset of* Γ *.* 0∈H *if and only if* $\chi_{H}\left(0\right)⊇\chi_{H}\left(\alpha \right)$ *for all* a∈Γ *.*

Proof.
Assume that 0∈H . So, $\chi_{H}\left(0\right)=\left[0,\;1\right]$ . Therefore, $\chi_{H}\left(0\right)⊇\chi_{H}\left(\alpha \right)$ for all α∈Γ .

Conversely, assume that $\chi_{H}\left(0\right)⊇\chi_{H}\left(\alpha \right)$ for all α∈Γ . Since H is nonempty subset of Γ , we have β∈H for β∈Γ . Therefore, $\chi_{H}\left(0\right)⊇\chi_{H}\left(\beta \right)=\left[0,\;1\right]$ . As a result, $\chi_{H}\left(0\right)=\left[0,\;1\right]$ . Hence, 0∈H .

Theorem 3.5 *A nonempty subset* H *of* Γ *is a subalgebra of* Γ *if and only if the characteristic hesitant fuzzy set* $\chi_{H}$ *is a hesitant fuzzy subalgebra of* Γ *.*

Proof.
Assume that H is subalgebra of Γ . Let α,β∈Γ .

(i) Here we will consider two cases.
  (a) Let α,β∈H . Clearly, $\chi_{H}\left(\alpha \right)=\left[0,\;1\right]$ and $\chi_{H}\left(\beta \right)=\left[0,\;1\right]$ . So, $\chi_{H}\left(\alpha \right)\cap \chi_{H}\left(\beta \right)=\left[0,\;1\right]$ . Since H is a subalgebra of Γ ; α⋆β∈H . As a result, $\chi_{H}\left(\alpha ⋆\beta \right)=\left[0,\;1\right]$ . Therefore, $\chi_{H}\left(\alpha ⋆\beta \right)⊇\chi_{H}\left(\alpha \right)\cap \chi_{H}\left(\beta \right)$ .
  (b) Let α∉H or β∉H . Then, $\chi_{H}\left(\alpha \right)=\emptyset$ or $\chi_{H}\left(\beta \right)=\emptyset$ . So, $\chi_{H}\left(\alpha \right)\cap \chi_{H}\left(\beta \right)=\emptyset$ . Clearly, $\chi_{H}\left(\alpha ⋆\beta \right)⊇\emptyset$ . Therefore, $\chi_{H}\left(\alpha ⋆\beta \right)⊇\chi_{H}\left(\alpha \right)\cap \chi_{H}\left(\beta \right)$ .
(ii) Here we will consider two cases.
  (a) Let α,β∈H . Clearly, $\chi_{H}\left(\alpha \right)=\left[0,\;1\right]$ and $\chi_{H}\left(\beta \right)=\left[0,\;1\right]$ . So, $\chi_{H}\left(\alpha \right)\cap \chi_{H}\left(\beta \right)=\left[0,\;1\right]$ . Since H is a subalgebra of Γ ; α+β∈H . As a result, $\chi_{H}\left(\alpha +\beta \right)=\left[0,\;1\right]$ . Therefore, $\chi_{H}\left(\alpha +\beta \right)⊇\chi_{H}\left(\alpha \right)\cap \chi_{H}\left(\beta \right)$ .
  (b) Let α∉H or β∉H . Clearly, $\chi_{H}\left(\alpha \right)=\emptyset$ or $\chi_{H}\left(\beta \right)=\emptyset$ . So, $\chi_{H}\left(\alpha \right)\cap \chi_{H}\left(\beta \right)=\emptyset$ . Clearly, $\chi_{H}\left(\alpha +\beta \right)⊇\emptyset$ . Therefore, $\chi_{H}\left(\alpha +\beta \right)⊇\chi_{H}\left(\alpha \right)\cap \chi_{H}\left(\beta \right)$ .
    Hence, $\chi_{H}$ is a hesitant fuzzy subalgebra of Γ.

Conversely, assume that $\chi_{H}$ is a hesitant fuzzy subalgebra of Γ. To show that H is a subalgebra of Γ .

(i) To show that 0∈H . Since $\chi_{H}\left(0\right)⊇\chi_{H}\left(\alpha \right)$ for all α∈Γ ; by theorem (3.4), 0∈H .
(ii) Let α,β∈H . Then, $\chi_{H}\left(\alpha \right)=\left[0,\;1\right]$ and $\chi_{H}\left(\beta \right)=\left[0,\;1\right]$ . Since $\chi_{H}$ is a hesitant fuzzy subalgebra of Γ ; $\chi_{H}\left(\alpha ⋆\beta \right)⊇\chi_{H}\left(\alpha \right)\cap \chi_{H}\left(\beta \right)=\left[0,\;1\right]$ . So, $\chi_{H}\left(\alpha ⋆\beta \right)=\left[0,\;1\right]$ . Hence, α⋆β∈H .
(iii) Let α,β∈H . Then, $\chi_{H}\left(\alpha \right)=\left[0,\;1\right]$ and $\chi_{H}\left(\beta \right)=\left[0,\;1\right]$ . Since $\chi_{H}$ is a hesitant fuzzy subalgebra of Γ ; $\chi_{H}\left(\alpha +\beta \right)⊇\chi_{H}\left(\alpha \right)\cap \chi_{H}\left(\beta \right)=\left[0,\;1\right]$ . So, $\chi_{H}\left(\alpha +\beta \right)=\left[0,\;1\right]$ . Hence, α+β∈H . Hence Γ is a subalgebra of Γ .

Let χ be a hesitant fuzzy set of Γ . For all ε∈P([0,1]) , define the level subsets of χ as $F^{⊇}\left(\chi ,\;\varepsilon \right)=\left\{\alpha \in \Gamma |\chi \left(a\right)⊇\varepsilon \right\}$ , $F^{⊃}\left(\chi ,\;\varepsilon \right)=\left\{\alpha \in \Gamma |\chi \left(a\right)⊃\varepsilon \right\}$ , $F^{\subseteq }\left(\chi ,\;\varepsilon \right)=\left\{\alpha \in \Gamma |\chi \left(a\right)\subseteq \varepsilon \right\}$ , and $F^{\subset }\left(\chi ,\;\varepsilon \right)=\left\{\alpha \in \Gamma |\chi \left(\alpha \right)\subset \varepsilon \right\}$ .

Theorem 3.6 *Let* χ *be a hesitant fuzzy set of* Γ *. Then* χ *is a hesitant fuzzy subalgebra of* Γ *if and only if for all* ε∈P([0,1]) *,* $F^{⊇}\left(\chi ,\;\varepsilon \right)\neq \emptyset$ *is a subalgebra of* Γ *.*

Proof.
Assume that χ is a hesitant fuzzy subalgebra of Γ . To show that $F^{⊇}\left(\chi ,\;\varepsilon \right)$ is a subalgebra of Γ . Let ε∈P([0,1]) be such that $F^{⊇}\left(\chi ,\;\varepsilon \right)\neq \emptyset$ .

(i) Let $\alpha \in F^{⊇}\left(\chi ,\;\varepsilon \right)$ . Then, χ(a)⊇ε . Since χ is a hesitant fuzzy subalgebra of Γ , implies that χ(0)⊇χ(α)⊇ε . Therefore, $0\in F^{⊇}\left(\chi ,\;\varepsilon \right)$ .
(ii) Let $\alpha ,\;\beta \in F^{⊇}\left(\chi ,\;\varepsilon \right)$ . Then, χ(α)⊇ε and χ(β)⊇ε . Since χ is a hesitant fuzzy subalgebra of Γ ; χ(α⋆β)⊇χ(α)∩χ(β)⊇ε . This implies that $\alpha ⋆\beta \in F^{⊇}\left(\chi ,\;\varepsilon \right)$ .
(iii) Let $\alpha ,\;\beta \in F^{⊇}\left(\chi ,\;\varepsilon \right)$ . Then, χ(α)⊇ε and χ(β)⊇ε . Since χ is a hesitant fuzzy subalgebra of Γ ; χ(α+β)⊇χ(α)∩χ(β)⊇ε . This implies that $\alpha +\beta \in F^{⊇}\left(\chi ,\;\varepsilon \right)$ . Hence, $F^{⊇}\left(\chi ,\;\varepsilon \right)$ is a subalgebra of Γ .

Conversely, assume that $F^{⊇}\left(\chi ,\;\varepsilon \right)$ is a subalgebra of Γ . To show that χ is a hesitant fuzzy subalgebra of Γ .

(i) Let α,β∈Γ . Take ε=χ(α)∩χ(β) . Therefore, χ(α)⊇ε and χ(β)⊇ε . As a result, $\alpha ,\;\beta \in F^{⊇}\left(\chi ,\;\varepsilon \right)\neq \emptyset$ . Since $F^{⊇}\left(\chi ,\;\varepsilon \right)$ is a subalgebra of Γ ; $\alpha ⋆\beta \in F^{⊇}\left(\chi ,\;\varepsilon \right)$ . Hence, χ(α⋆β)⊇ε=χ(α)∩χ(β) .
(ii) Let α,β∈Γ . Take ε=χ(α)∩χ(β) . Therefore, χ(α)⊇ε and χ(β)⊇ε . As a result, $\alpha ,\;\beta \in F^{⊇}\left(\chi ,\;\varepsilon \right)\neq \emptyset$ . Since $F^{⊇}\left(\chi ,\;\varepsilon \right)$ is a subalgebra of Γ ; $\alpha +\beta \in F^{⊇}\left(\chi ,\;\varepsilon \right)$ . Hence, χ(α+β)⊇ε=χ(α)∩χ(β) .

Hence, χ is a hesitant fuzzy subalgebra of Γ .

Theorem 3.7 *Let* χ *be a hesitant fuzzy set of* Γ *. If* Im(χ) *is a chain and for all* ε∈P([0,1]) *,* $F^{⊃}\left(\chi ,\;\varepsilon \right)\neq \emptyset$ *is a subalgebra of* Γ *, then* χ *is a hesitant fuzzy subalgebra of* Γ *.*

Proof.
Assume that Im(χ) is a chain and for all ε∈P([0,1]) , a nonempty subset $F^{⊃}\left(\chi ,\;\varepsilon \right)$ is a subalgebra of Γ . To show that χ is a hesitant fuzzy subalgebra of Γ .

(i) Let α,β∈Γ . To show that χ(α+β)⊇χ(α)∩χ(β) . Suppose that χ(α+β)⊉χ(α)∩χ(β) . Since Im(χ) is a chain; implies that χ(α+β)⊂χ(α)∩χ(β) . Take ε=χ(α+β) . Therefore, χ(α)⊃ε and χ(β)⊃ε . As a result, $\alpha ,\;\beta \in F^{⊃}\left(\chi ,\;\varepsilon \right)\neq \emptyset$ . Since $F^{⊃}\left(\chi ,\;\varepsilon \right)$ is a subalgebra of Γ ; $\alpha +\beta \in F^{⊃}\left(\chi ,\;\varepsilon \right)$ . So, χ(α+β)⊃ε=χ(α+β) . This is a contradiction. Thus, χ(α+β)⊇χ(α)∩χ(β) for all α,β∈Γ .
(ii) Let α,β∈Γ . To show that χ(α⋆β)⊇χ(α)∩χ(β) . Suppose that χ(α⋆β)⊉χ(α)∩χ(β) . Since Im(χ) is a chain; implies that χ(α⋆β)⊂χ(α)∩χ(β) . Take ε=χ(α⋆β) . Therefore, χ(α)⊃ε and χ(β)⊃ε . As a result, $\alpha ,\;\beta \in F^{⊃}\left(\chi ,\;\varepsilon \right)\neq \emptyset$ . Since $F^{⊃}\left(\chi ,\;\varepsilon \right)$ is a subalgebra of Γ ; $\alpha ⋆\beta \in F^{⊃}\left(\chi ,\;\varepsilon \right)$ . So, χ(α⋆β)⊃ε=χ(α⋆β) . This is a contradiction. Thus, χ(α⋆β)⊇χ(α)∩χ(β) for all α,β∈Γ .

Hence, χ is a hesitant fuzzy subalgebra of Γ .

Theorem 3.8 *Let* $\bar{\chi }$ *be a hesitant fuzzy set of* Γ *. Then* $\bar{\chi }$ *is a hesitant fuzzy subalgebra of* Γ *if and only if for all* ε∈P([0,1]) *,* $F^{\subseteq }\left(\chi ,\;\varepsilon \right)\neq \emptyset$ *is a subalgebra of* Γ *.*

Proof.
Assume that $\bar{\chi }$ is a hesitant fuzzy subalgebra of Γ . To show that $F^{\subseteq }\left(\chi ,\;\varepsilon \right)$ is a subalgebra of Γ . Let ε∈P([0,1]) be such that $F^{\subseteq }\left(\chi ,\;\varepsilon \right)\neq \emptyset$ .

(i) Let $\alpha \in F^{\subseteq }\left(\chi ,\;\varepsilon \right)$ . Then, χ(α)⊆ε . Since $\bar{\chi }$ is a hesitant fuzzy subalgebra of Γ , implies that $\bar{\chi }\left(0\right)⊇\bar{\chi }\left(\alpha \right)$ . Clearly, [0,1]\χ(0)⊇[0,1]\χ(α) . Therefore, χ(0)⊆χ(α)⊆ε . Therefore, $0\in F^{\subseteq }\left(\chi ,\;\varepsilon \right)$ .
(ii) Let $\alpha ,\;\beta \in F^{\subseteq }\left(\chi ,\;\varepsilon \right)$ . Then, χ(α)⊆ε and χ(β)⊆ε . Since $\bar{\chi }$ is a hesitant fuzzy subalgebra of Γ ; $\bar{\chi }\left(\alpha +\beta \right)⊇\bar{\chi }\left(\alpha \right)\cap \bar{\chi }\left(\beta \right)$ . So, [0,1]\χ(α+β)⊇([0,1]\χ(α))∩([0,1]\χ(β))=([0,1]\(χ(α)∪χ(β)) . Therefore, χ(α+β)⊆χ(α)∪χ(β)⊆ε . Hence, $\alpha +\beta \in F^{\subseteq }\left(\chi ,\;\varepsilon \right)$ .
(iii) Let $\alpha ,\;\beta \in F^{\subseteq }\left(\chi ,\;\varepsilon \right)$ . Then, χ(α)⊆ε and χ(β)⊆ε . Since $\bar{\chi }$ is a hesitant fuzzy subalgebra of Γ ; $\bar{\chi }\left(\alpha ⋆\beta \right)⊇\bar{\chi }\left(\alpha \right)\cap \bar{\chi }\left(\beta \right)$ . So, [0,1]\χ(α⋆β)⊇([0,1]\χ(α))∩([0,1]\χ(β))=([0,1]\(χ(α)∪χ(β)) . Therefore, χ(α⋆β)⊆χ(α)∪χ(β)⊆ε . Hence, $\alpha ⋆\beta \in F^{\subseteq }\left(\chi ,\;\varepsilon \right)$ . Therefore, $F^{\subseteq }\left(\chi ,\;\varepsilon \right)$ is a subalgebra of Γ .

Conversely, assume that $F^{\subseteq }\left(\chi ,\;\varepsilon \right)$ is a subalgebra of Γ . To show that $\bar{\chi }$ is a hesitant fuzzy subalgebra of Γ .

(i) Let α,β∈Γ . Take ε=χ(α)∪χ(β) . Therefore, χ(α)⊆ε and χ(β)⊆ε . As a result, $\alpha ,\;\beta \in F^{\subseteq }\left(\chi ,\;\varepsilon \right)\neq \emptyset$ . Since $F^{\subseteq }\left(\chi ,\;\varepsilon \right)$ is a subalgebra of Γ ; $\alpha ⋆\beta \in F^{\subseteq }\left(\chi ,\;\varepsilon \right)$ . Clearly, χ(α⋆β)⊆ε=χ(α)∪χ(β) . As a result, $\left[0,\;1\right]\backslash \chi \left(\alpha ⋆\beta \right)⊇\left[0,\;1\right]\backslash \left(\chi \left(\alpha \right)\cup \chi \left(\beta \right)\right)=\left(\left[0,\;1\right]\backslash \chi \left(\alpha \right)\right)\cap \left(\left[0,\;1\right]\backslash \chi \left(\beta \right)\right)=\bar{\chi }\left(\alpha \right)\cap \bar{\chi }\left(\beta \right)$ . Therefore, $\bar{\chi }\left(\alpha ⋆\beta \right)⊇\bar{\chi }\left(\alpha \right)\cap \bar{\chi }\left(\beta \right)$ .
(ii) Let α,β∈Γ . Take ε=χ(α)∪χ(β) . Therefore, χ(α)⊆ε and χ(β)⊆ε . As a result, $\alpha ,\;\beta \in F^{\subseteq }\left(\chi ,\;\varepsilon \right)\neq \emptyset$ . Since $F^{\subseteq }\left(\chi ,\;\varepsilon \right)$ is a subalgebra of Γ ; $\alpha +\beta \in F^{\subseteq }\left(\chi ,\;\varepsilon \right)$ . Clearly, χ(α+β)⊆ε=χ(α)∪χ(β) . As a result, $\left[0,\;1\right]\backslash \chi \left(\alpha +\beta \right)⊇\left[0,\;1\right]\backslash \left(\chi \left(\alpha \right)\cup \chi \left(\beta \right)\right)=\left(\left[0,\;1\right]\backslash \chi \left(\alpha \right)\right)\cap \left(\left[0,\;1\right]\backslash \chi \left(\beta \right)\right)=\bar{\chi }\left(\alpha \right)\cap \bar{\chi }\left(\beta \right)$ . Therefore, $\bar{\chi }\left(\alpha +\beta \right)⊇\bar{\chi }\left(\alpha \right)\cap \bar{\chi }\left(\beta \right)$ .

Hence, $\bar{\chi }$ is a hesitant fuzzy subalgebra of Γ .

Theorem 3.9 *Let* χ *be a hesitant fuzzy set of* Γ *. If* Im(χ) *is a chain and for all* ε∈P([0,1]) *,* $F^{\subset }\left(\chi ,\;\varepsilon \right)\neq \emptyset$ *is a subalgebra of* Γ *, then* $\bar{\chi }$ *is a hesitant fuzzy subalgebra of* Γ *.*

Proof.
Assume that Im(χ) is a chain and for all ε∈P([0,1]) , a nonempty subset $F^{\subset }\left(\chi ,\;\varepsilon \right)$ is a subalgebra of Γ . To show that $\bar{\chi }$ is a hesitant fuzzy subalgebra of Γ .

(i) Let α,β∈Γ . To show that $\bar{\chi }\left(\alpha +\beta \right)⊇\bar{\chi }\left(\alpha \right)\cap \bar{\chi }\left(\beta \right)$ . Suppose that $\bar{\chi }\left(\alpha +\beta \right)⊉\bar{\chi }\left(\alpha \right)\cap \bar{\chi }\left(\beta \right)$ . Since Im(χ) is a chain; implies that $\bar{\chi }\left(\alpha +\beta \right)\subset \bar{\chi }\left(\alpha \right)\cap \bar{\chi }\left(\beta \right)$ . Clearly, [0,1]\χ(α+β)⊂([0,1]\χ(α))∩([0,1]\χ(β))=[0,1]\(χ(α)∪χ(β)) . So, χ(α+β)⊃χ(α)∪χ(β) . Take ε=χ(α+β) . Therefore, χ(α)⊂ε and χ(β)⊂ε . As a result, $\alpha ,\;\beta \in F^{\subset }\left(\chi ,\;\varepsilon \right)\neq \emptyset$ . Since $F^{\subset }\left(\chi ,\;\varepsilon \right)$ is a subalgebra of Γ ; $\alpha +\beta \in F^{\subset }\left(\chi ,\;\varepsilon \right)$ . So, χ(α+β)⊂ε=χ(α+β) . This is a contradiction. Thus, $\bar{\chi }\left(\alpha +\beta \right)⊇\bar{\chi }\left(\alpha \right)\cap \bar{\chi }\left(\beta \right)$ for all α,β∈Γ .
(ii) Let α,β∈Γ . To show that $\bar{\chi }\left(\alpha ⋆\beta \right)⊇\bar{\chi }\left(\alpha \right)\cap \bar{\chi }\left(\beta \right)$ . Suppose that $\bar{\chi }\left(\alpha ⋆\beta \right)⊉\bar{\chi }\left(\alpha \right)\cap \bar{\chi }\left(\beta \right)$ . Since Im(χ) is a chain; implies that $\bar{\chi }\left(\alpha ⋆\beta \right)\subset \bar{\chi }\left(\alpha \right)\cap \bar{\chi }\left(\beta \right)$ . Clearly, [0,1]\χ(α⋆β)⊂([0,1]\χ(α))∩([0,1]\χ(β))=[0,1]\(χ(α)∪χ(β)) . So, χ(α⋆β)⊃χ(α)∪χ(β) . Take ε=χ(α⋆β) . Therefore, χ(α)⊂ε and χ(β)⊂ε . As a result, $\alpha ,\;\beta \in F^{\subset }\left(\chi ,\;\varepsilon \right)\neq \emptyset$ . Since $F^{\subset }\left(\chi ,\;\varepsilon \right)$ is a subalgebra of Γ ; $\alpha ⋆\beta \in F^{\subset }\left(\chi ,\;\varepsilon \right)$ . So, χ(α⋆β)⊂ε=χ(α⋆β) . This is a contradiction. Thus, $\bar{\chi }\left(\alpha ⋆\beta \right)⊇\bar{\chi }\left(\alpha \right)\cap \bar{\chi }\left(\beta \right)$ for all α,β∈Γ . Hence, $\bar{\chi }$ is a hesitant fuzzy subalgebra of Γ .

## 4. Hesitant Fuzzy Ideals of Autometrized Algebra

This section presents the concept of hesitant fuzzy ideals in autometrized algebras and explores several fundamental properties related to these ideals.

Definition 4.1 *A hesitant fuzzy subset* χ *of* Γ *is called a hesitant fuzzy ideal of* Γ *if for all* α,β∈Γ *;*

(i)
χ(α+β)⊇χ(α)∩χ(β) .
(ii) if α⋆0≼β⋆0 , then χ(α)⊇χ(β) .

Example 4.2 *Let* Γ={0,α,β,γ} *with* 0≼α,β≼γ *and elements* α,β *are incomparable. Define* ⋆ *and* + *by the following tables.*

**Table:** 

⋆	0	α	β	γ
0	0	α	β	γ
α	α	0	γ	β
β	β	γ	0	α
γ	γ	β	α	0

**Table:** 

+	0	α	β	γ
0	0	α	β	γ
α	α	α	γ	γ
β	β	γ	β	γ
γ	γ	γ	γ	γ

*Then,* Γ *is an autometrized algebra. Define a hesitant fuzzy subset* χ:Γ→P([0,1]) *of* Γ *by:*

$$\chi \left(\delta \right)=\left\{ \begin{array}{ll} \left\{0.2,\;0.5\right\}, & \text{if}\;\alpha \in \left\{0,\;\alpha \right\}.\\ \left\{0.2\right\}, & \text{otherwise}. \end{array}\right.$$

*Now, check the two closure properties,*

(i)
$$\begin{array}{l} \chi \left(0+\alpha \right)=\chi \left(\alpha \right)=\left\{0.2,0.5\right\}⊇\chi \left(0\right)\cap \chi \left(\alpha \right)=\left\{0.2,0.5\right\}\\ \chi \left(0+\beta \right)=\chi \left(\beta \right)=\left\{0.2\right\}⊇\chi \left(0\right)\cap \chi \left(\beta \right)=\left\{0.2\right\}\\ \chi \left(0+\gamma \right)=\chi \left(\gamma \right)=\left\{0.2\right\}⊇\chi \left(0\right)\cap \chi \left(\gamma \right)=\left\{0.2\right\}\\ \chi \left(\alpha +\alpha \right)=\chi \left(\alpha \right)=\left\{0.2,0.5\right\}⊇\chi \left(\alpha \right)\cap \chi \left(\alpha \right)=\left\{0.2,0.5\right\}\\ \chi \left(\alpha +\beta \right)=\chi \left(\gamma \right)=\left\{0.2\right\}⊇\chi \left(\alpha \right)\cap \chi \left(\beta \right)=\left\{0.2\right\}\\ \chi \left(\alpha +\gamma \right)=\chi \left(\gamma \right)=\left\{0.2\right\}⊇\chi \left(\alpha \right)\cap \chi \left(\gamma \right)=\left\{0.2\right\}\\ \chi \left(\beta +\beta \right)=\chi \left(\beta \right)=\left\{0.2\right\}⊇\chi \left(\beta \right)\cap \chi \left(\beta \right)=\left\{0.2\right\}\\ \chi \left(\beta +\gamma \right)=\chi \left(\gamma \right)=\left\{0.2\right\}⊇\chi \left(\beta \right)\cap \chi \left(\gamma \right)=\left\{0.2\right\}\\ \chi \left(\gamma +\gamma \right)=\chi \left(\gamma \right)=\left\{0.2\right\}⊇\chi \left(\gamma \right)\cap \chi \left(\gamma \right)=\left\{0.2\right\} \end{array}$$
(ii)
$$\begin{array}{l} 0⋆0≼\alpha ⋆0\Rightarrow \chi \left(0\right)=\left\{0.2,0.5\right\}⊇\chi \left(\alpha \right)=\left\{0.2,0.5\right\}\\ 0⋆0≼\beta ⋆0\Rightarrow \chi \left(0\right)=\left\{0.2,0.5\right\}⊇\chi \left(\beta \right)=\left\{0.2\right\}\\ 0⋆0≼\gamma ⋆0\Rightarrow \chi \left(0\right)=\left\{0.2,0.5\right\}⊇\chi \left(\gamma \right)=\left\{0.2\right\}\\ \alpha ⋆0≼\gamma ⋆0\Rightarrow \chi \left(\alpha \right)=\left\{0.2,0.5\right\}⊇\chi \left(\gamma \right)=\left\{0.2\right\}\\ \beta ⋆0≼\gamma ⋆0\Rightarrow \chi \left(\beta \right)=\left\{0.2\right\}⊇\chi \left(\gamma \right)=\left\{0.2\right\} \end{array}$$

*Therefore,* χ *is a hesitant fuzzy ideal of* Γ *.*

Lemma 4.3 *If* χ *is a hesitant fuzzy ideal of* Γ *, then for* α∈Γ *;* χ(0)⊇χ(α) *.*

Proof.
Assume that χ is a hesitant fuzzy ideal of Γ . Since 0⋆0≼α⋆0 ; implies that that χ(0)⊇χ(α) .

Lemma 4.4 *Let* Γ *be a normal autometrized algebra. If* χ *is a hesitant fuzzy ideal of* Γ *, then for* α∈Γ *;* χ(α⋆0)=χ(0) *.*

Proof.
Assume that χ is a hesitant fuzzy ideal of Γ . Since Γ is normal; implies that (α⋆0)⋆0=α⋆0 . This implies that (α⋆0)⋆0≼α⋆0 and α⋆0≼(α⋆0)⋆0 ; implies that χ(α⋆0)⊇χ(α) and χ(α)⊇χ(α⋆0) . Therefore, χ(α⋆0)=χ(α) .

Theorem 4.5 *Let* Γ *be a normal autometrized algebra. Every hesitant fuzzy ideal of* Γ *is a hesitant fuzzy subalgebra of* Γ *.*

Proof.
Assume that χ is a hesitant fuzzy ideal of Γ . Let α,β∈Γ .

(i) By the definition of ideal, χ(α+β)⊇χ(α)∩χ(β) .
(ii) Now, to show that χ(α⋆β)⊇χ(α)∩χ(β) . Since Γ is normal; (α⋆β)⋆0≼α⋆0+β⋆0≼(α⋆0+β⋆0)⋆0 . Therefore,
$$\begin{array}{l} \chi \left(\alpha ⋆\beta \right)⊇\chi \left(\alpha ⋆0+\beta ⋆0\right)\\ \;⊇\chi \left(\alpha ⋆0\right)\cap \chi \left(\beta ⋆0\right)\\ \;⊇\chi \left(\alpha \right)\cap \chi (\beta )\text{[by Lemma (4.4)]} \end{array}$$

Therefore, χ(α⋆β)⊇χ(α)∩χ(β) . Hence, χ is a hesitant fuzzy subalgebra of Γ .

Theorem 4.6 *A nonempty subset* H *of* Γ *is an ideal of* Γ *if and only if the characteristic hesitant fuzzy set* $\chi_{H}$ *is a hesitant fuzzy ideal of* Γ *.*

Proof.
Assume that H is an ideal of Γ . Let α,β∈Γ .

(i) Here we will consider two cases.
  (a) Let α,β∈H . Clearly, $\chi_{H}\left(\alpha \right)=\left[0,\;1\right]$ and $\chi_{H}\left(\beta \right)=\left[0,\;1\right]$ . So, $\chi_{H}\left(\alpha \right)\cap \chi_{H}\left(\beta \right)=\left[0,\;1\right]$ . Since H is an ideal of Γ ; α+β∈H . As a result, $\chi_{H}\left(\alpha +\beta \right)=\left[0,\;1\right]$ . Therefore, $\chi_{H}\left(\alpha +\beta \right)⊇\chi_{H}\left(\alpha \right)\cap \chi_{H}\left(\beta \right)$ .
  (b) Let α∉H or β∉H . Then, $\chi_{H}\left(\alpha \right)=\emptyset$ or $\chi_{H}\left(\beta \right)=\emptyset$ . So, $\chi_{H}\left(\alpha \right)\cap \chi_{H}\left(\beta \right)=\emptyset$ . Clearly, $\chi_{H}\left(\alpha +\beta \right)⊇\emptyset$ . Therefore, $\chi_{H}\left(\alpha +\beta \right)⊇\chi_{H}\left(\alpha \right)\cap \chi_{H}\left(\beta \right)$ .
(ii) Suppose α⋆0≼β⋆0 . To show that $\chi_{H}\left(\alpha \right)⊇\chi_{H}\left(\beta \right)$ . Here we will consider two cases.
  (a) Let β∈H . Clearly, $\chi_{H}\left(\beta \right)=\left[0,\;1\right]$ . Since H is an ideal; α∈H . As a result, $\chi_{H}\left(\alpha \right)=\left[0,\;1\right]$ . Hence, $\chi_{H}\left(\alpha \right)⊇\chi_{H}\left(\beta \right)$ .
  (b) Let α∉H or β∉H . Clearly, $\chi_{H}\left(\alpha \right)=\emptyset$ or $\chi_{H}\left(\beta \right)=\emptyset$ . Therefore, $\chi_{H}\left(\alpha \right)⊇\chi_{H}\left(\beta \right)=\emptyset$ .

Hence, $\chi_{H}$ is a hesitant fuzzy ideal of Γ .

Conversely, assume that $\chi_{H}$ is a hesitant fuzzy ideal of Γ . To show that H is an ideal of Γ .

(i) Let α,β∈H . Then, $\chi_{H}\left(\alpha \right)=\left[0,\;1\right]$ and $\chi_{H}\left(\beta \right)=\left[0,\;1\right]$ . Since $\chi_{H}$ is a hesitant fuzzy ideal of Γ ; $\chi_{H}\left(\alpha +\beta \right)⊇\chi_{H}\left(\alpha \right)\cap \chi_{H}\left(\beta \right)=\left[0,\;1\right]$ . So, $\chi_{H}\left(\alpha +\beta \right)=\left[0,\;1\right]$ . Hence, α+β∈H .
(ii) Let α,β∈Γ . Suppose α⋆0≼β⋆0 . Let β∈H . Clearly, $\chi_{H}\left(\beta \right)=\left[0,\;1\right]$ . To show that α∈H . Since $\chi_{H}$ is a hesitant fuzzy ideal of Γ , $\chi_{H}\left(\alpha \right)⊇\chi_{H}\left(\beta \right)=\left[0,\;1\right]$ . Therefore, $\chi_{H}\left(\alpha \right)=\left[0,\;1\right]$ . Thus, α∈H . Hence, H is an ideal of Γ .

Theorem 4.7 *Let* χ *be a hesitant fuzzy set of* Γ *. Then* χ *is a hesitant fuzzy ideal of* Γ *if and only if for all* ε∈P([0,1]) *, a nonempty subset* $F^{⊇}\left(\chi ,\;\varepsilon \right)$ *is an ideal of* Γ *.*

Proof.
Assume that χ is a hesitant fuzzy ideal of Γ . To show that $F^{⊇}\left(\chi ,\;\varepsilon \right)$ is an ideal of Γ . Let ε∈P([0,1]) be such that $F^{⊇}\left(\chi ,\;\varepsilon \right)\neq \emptyset$ .

(i) Let $\alpha ,\;\beta \in F^{⊇}\left(\chi ,\;\varepsilon \right)$ . Then, χ(α)⊇ε and χ(β)⊇ε . Since χ is a hesitant fuzzy ideal of Γ ; χ(α+β)⊇χ(α)∩χ(β)⊇ε . This implies that $\alpha +\beta \in F^{⊇}\left(\chi ,\;\varepsilon \right)$ .
(ii) Let α,β∈Γ . Suppose α⋆0≼β⋆0 . Let $\beta \in F^{⊇}\left(\chi ,\;\varepsilon \right)$ . Clearly, χ(β)⊇ε . To show that $\alpha \in F^{⊇}\left(\chi ,\;\varepsilon \right)$ . Since χ is a hesitant fuzzy ideal of Γ , χ(α)⊇χ(β)⊇ε . Therefore, χ(α)⊇ε . Thus, $\alpha \in F^{⊇}\left(\chi ,\;\varepsilon \right)$ . Hence, $F^{⊇}\left(\chi ,\;\varepsilon \right)$ is an ideal of Γ .

Conversely, assume that $F^{⊇}\left(\chi ,\;\varepsilon \right)$ is an ideal of Γ . To show that χ is a hesitant fuzzy ideal of Γ .

(i) Let α,β∈Γ . Take ε=χ(α)∩χ(β) . Therefore, χ(α)⊇ε and χ(β)⊇ε . As a result, $\alpha ,\;\beta \in F^{⊇}\left(\chi ,\;\varepsilon \right)\neq \emptyset$ . Since $F^{⊇}\left(\chi ,\;\varepsilon \right)$ is an ideal of Γ ; $\alpha +\beta \in F^{⊇}\left(\chi ,\;\varepsilon \right)$ . Hence, χ(α+β)⊇ε=χ(α)∩χ(β) .
(ii) Let α,β∈Γ . Suppose α⋆0≼β⋆0 . Take ε=χ(β) . So, $\beta \in F^{⊇}\left(\chi ,\;\varepsilon \right)$ . Since $F^{⊇}\left(\chi ,\;\varepsilon \right)$ is an ideal; $\alpha \in F^{⊇}\left(\chi ,\;\varepsilon \right)$ . As a result, χ(α)⊇ε=χ(β) .

Hence, χ is a hesitant fuzzy ideal of Γ .

Theorem 4.8 *Let* χ *be a hesitant fuzzy set of* Γ *. If* Im(χ) *is a chain and for all* ε∈P([0,1]) *, a nonempty subset* $F^{⊃}\left(\chi ,\;\varepsilon \right)$ *is an ideal of* Γ *, then* χ *is a hesitant fuzzy ideal of* Γ *.*

Proof.
Assume that Im(χ) is a chain and for all ε∈P([0,1]) , a nonempty subset $F^{⊃}\left(\chi ,\;\varepsilon \right)$ is an ideal of Γ . To show that χ is a hesitant fuzzy ideal of Γ .

(i) Let α,β∈Γ . To show that χ(α+β)⊇χ(α)∩χ(β) . Suppose that χ(α+β)⊉χ(α)∩χ(β) . Since Im(χ) is a chain; implies that χ(α+β)⊂χ(α)∩χ(β) . Then, χ(α+β)∈P([0,1]) . Take ε=χ(α+β) . Therefore, χ(α)⊃ε and χ(β)⊃ε . As a result, $\alpha ,\;\beta \in F^{⊃}\left(\chi ,\;\varepsilon \right)\neq \emptyset$ . Since $F^{⊃}\left(\chi ,\;\varepsilon \right)$ is an ideal of Γ ; $\alpha +\beta \in F^{⊃}\left(\chi ,\;\varepsilon \right)$ . So, χ(α+β)⊃ε=χ(α+β) . This is a contradiction. Thus, χ(α+β)⊇χ(α)∩χ(β) for all α,β∈Γ .
(ii) Let α,β∈Γ . Suppose α⋆0≼β⋆0 . To show that χ(α)⊇χ(β) . Suppose that χ(α)⊉χ(β) . Since Im(χ) is a chain; implies that χ(α)⊂χ(β) . Then, χ(α)∈P([0,1]) . Take ε=χ(α) . Therefore, χ(β)⊃ε . As a result, $\beta \in F^{⊃}\left(\chi ,\;\varepsilon \right)$ . Since $F^{⊃}\left(\chi ,\;\varepsilon \right)$ is an ideal of Γ ; $\alpha \in F^{⊃}\left(\chi ,\;\varepsilon \right)$ . So, χ(α)⊃ε=χ(α) . This is a contradiction. Therefore, χ(α)⊇(β) for all α,β∈Γ .

Hence, χ is a hesitant fuzzy ideal of Γ .

Theorem 4.9 *Let* $\bar{\chi }$ *be a hesitant fuzzy set of* Γ *. Then* $\bar{\chi }$ *is a hesitant fuzzy ideal of* Γ *if and only if for all* ε∈P([0,1]) *, a nonempty subset* $F^{\subseteq }\left(\chi ,\;\varepsilon \right)$ *is an ideal of* Γ *.*

Proof.
Assume that $\bar{\chi }$ is a hesitant fuzzy ideal of Γ . To show that $F^{\subseteq }\left(\chi ,\;\varepsilon \right)$ is an ideal of Γ . Let ε∈P([0,1]) be such that $F^{\subseteq }\left(\chi ,\;\varepsilon \right)\neq \emptyset$ .

(i) Let $\alpha \in F^{\subseteq }\left(\chi ,\;\varepsilon \right)$ . Then, χ(α)⊆ε . Since $\bar{\chi }$ is a hesitant fuzzy ideal of Γ , implies that $\bar{\chi }\left(0\right)⊇\bar{\chi }\left(\alpha \right)$ . Clearly, [0,1]\χ(0)⊇[0,1]\χ(α) . Therefore, χ(0)⊆χ(α)⊆ε . Therefore, $0\in F^{\subseteq }\left(\chi ,\;\varepsilon \right)$ .
(ii) Let $\alpha ,\;\beta \in F^{\subseteq }\left(\chi ,\;\varepsilon \right)$ . Then, χ(α)⊆ε and χ(β)⊆ε . Since $\bar{\chi }$ is a hesitant fuzzy ideal of Γ ; $\bar{\chi }\left(\alpha +\beta \right)⊇\bar{\chi }\left(\alpha \right)\cap \bar{\chi }\left(\beta \right)$ . So, [0,1]\χ(α+β)⊇([0,1]\χ(α))∩([0,1]\χ(β))=([0,1]\(χ(α)∪χ(β)) . Therefore, χ(α+β)⊆χ(α)∪χ(β)⊆ε . Hence, $\alpha +\beta \in F^{\subseteq }\left(\chi ,\;\varepsilon \right)$ .
(iii) Let α,β∈Γ . Suppose α⋆0≼β⋆0 . Let $\beta \in F^{\subseteq }\left(\chi ,\;\varepsilon \right)$ . Clearly, χ(β)⊆ε . To show that $\alpha \in F^{\subseteq }\left(\chi ,\;\varepsilon \right)$ . Since $\bar{\chi }$ is a hesitant fuzzy ideal of Γ , $\bar{\chi }\left(\alpha \right)⊇\bar{\chi }\left(\beta \right)$ . So, [0,1]\χ(α)⊇[0,1]\χ(β) . Therefore, χ(α)⊆χ(β)⊆ε . Thus, $\alpha \in F^{\subseteq }\left(\chi ,\;\varepsilon \right)$ . Hence, $F^{\subseteq }\left(\chi ,\;\varepsilon \right)$ is an ideal of Γ .

Conversely, assume that $F^{\subseteq }\left(\chi ,\;\varepsilon \right)$ is an ideal of Γ . To show that $\bar{\chi }$ is a hesitant fuzzy ideal of Γ .

(i) Let α,β∈Γ . Take ε=χ(α)∪χ(β) . Therefore, χ(α)⊆ε and χ(β)⊆ε . As a result, $\alpha ,\;\beta \in F^{\subseteq }\left(\chi ,\;\varepsilon \right)\neq \emptyset$ . Since $F^{\subseteq }\left(\chi ,\;\varepsilon \right)$ is an ideal of Γ ; $\alpha +\beta \in F^{\subseteq }\left(\chi ,\;\varepsilon \right)$ . Clearly, χ(α+β)⊆ε=χ(α)∪χ(β) . As a result, $\left[0,\;1\right]\backslash \chi \left(\alpha +\beta \right)⊇\left[0,\;1\right]\backslash \left(\chi \left(\alpha \right)\cup \chi \left(\beta \right)\right)=\left(\left[0,\;1\right]\backslash \chi \left(\alpha \right)\right)\cap \left(\left[0,\;1\right]\backslash \chi \left(\beta \right)\right)=\bar{\chi }\left(\alpha \right)\cap \bar{\chi }\left(\beta \right)$ . Therefore, $\bar{\chi }\left(\alpha +\beta \right)⊇\bar{\chi }\left(\alpha \right)\cap \bar{\chi }\left(\beta \right)$ .
(ii) Let α,β∈Γ . Suppose α⋆0≼β⋆0 . Take ε=χ(β) . So, $\beta \in F^{\subseteq }\left(\chi ,\;\varepsilon \right)$ . Since $F^{\subseteq }\left(\chi ,\;\varepsilon \right)$ is an ideal; $\alpha \in F^{\subseteq }\left(\chi ,\;\varepsilon \right)$ . As a result, χ(α)⊆ε=χ(β) . Clearly, [0,1]\χ(α)⊇[0,1]\χ(β) . Therefore, $\bar{\chi }\left(\alpha \right)⊇\bar{\chi }\left(\beta \right)$ . Hence, $\bar{\chi }$ is a hesitant fuzzy ideal of Γ .

Theorem 4.10 *Let* χ *be a hesitant fuzzy set of* Γ *. If* Im(χ) *is a chain and for all* ε∈P([0,1]) *, a nonempty subset* $F^{\subset }\left(\chi ,\;\varepsilon \right)$ *is an ideal of* Γ *, then* $\bar{\chi }$ *is a hesitant fuzzy ideal of* Γ *.*

Proof.
Assume that Im(χ) is a chain and for all ε∈P([0,1]) , a nonempty subset $F^{\subset }\left(\chi ,\;\varepsilon \right)$ is an ideal of Γ . To show that $\bar{\chi }$ is a hesitant fuzzy ideal of Γ .

(i) Let α,β∈Γ . To show that $\bar{\chi }\left(\alpha +\beta \right)⊇\bar{\chi }\left(\alpha \right)\cap \bar{\chi }\left(\beta \right)$ . Suppose that $\bar{\chi }\left(\alpha +\beta \right)⊉\bar{\chi }\left(\alpha \right)\cap \bar{\chi }\left(\beta \right)$ . Since Im(χ) is a chain; implies that $\bar{\chi }\left(\alpha +\beta \right)\subset \bar{\chi }\left(\alpha \right)\cap \bar{\chi }\left(\beta \right)$ . Clearly, [0,1]\χ(α+β)⊂([0,1]\χ(α))∩([0,1]\χ(β))=[0,1]\(χ(α)∪χ(β)) . So, χ(α+β)⊃χ(α)∪χ(β) . Take ε=χ(α+β) . Therefore, χ(α)⊂ε and χ(β)⊂ε . As a result, $\alpha ,\;\beta \in F^{\subset }\left(\chi ,\;\varepsilon \right)\neq \emptyset$ . Since $F^{\subset }\left(\chi ,\;\varepsilon \right)$ is an ideal of Γ ; $\alpha +\beta \in F^{\subset }\left(\chi ,\;\varepsilon \right)$ . So, χ(α+β)⊂ε=χ(α+β) . This is a contradiction. Thus, $\bar{\chi }\left(\alpha +\beta \right)⊇\bar{\chi }\left(\alpha \right)\cap \bar{\chi }\left(\beta \right)$ for all α,β∈Γ .
(ii) Let α,β∈Γ . Suppose α⋆0≼β⋆0 . To show that $\bar{\chi }\left(\alpha \right)⊇\bar{\chi }\left(\beta \right)$ . Suppose that $\bar{\chi }\left(\alpha \right)⊉\bar{\chi }\left(\beta \right)$ . Since Im(χ) is a chain; implies that $\bar{\chi }\left(\alpha \right)\subset \bar{\chi }\left(\beta \right)$ . Then, [0,1]\χ(a)⊂[0,1]\χ(b) . Clearly, χ(α)⊃χ(β) . Take ε=χ(α) . Therefore, χ(β)⊂ε . As a result, $\beta \in F^{\subset }\left(\chi ,\;\varepsilon \right)$ . Since $F^{\subset }\left(\chi ,\;\varepsilon \right)$ is an ideal of Γ ; $\alpha \in F^{\subset }\left(\chi ,\;\varepsilon \right)$ . So, χ(α)⊂ε=χ(α) . This is a contradiction. Therefore, $\bar{\chi }\left(\alpha \right)⊇\bar{\chi }\left(\beta \right)$ for all α,β∈Γ . Hence, $\bar{\chi }$ is a hesitant fuzzy ideal of Γ .

## 5. Hesitant Fuzzy Congruence on Autometrized Algebra

In this section, we introduce the hesitant fuzzy congruence relation of autometrized algebras and examine some related properties.

Definition 5.1 *A hesitant fuzzy relation on* Γ *is a mapping* Ψ:Γ×Γ→P([0,1]) *, where* P([0,1]) *means the power set of* [0,1] *.*

Definition 5.2 *Let* Ψ *be a hesitant fuzzy relation on* Γ *. Then,* $\bar{\Psi }\left(a,\;b\right)=P\left([0,1]\right)\backslash \Psi \left(a,\;b\right)$ *for all* a,b∈Γ *which is said to be the complement of* Ψ *on* Γ *.*

Definition 5.3 *If* Ψ *is a hesitant fuzzy equivalent relation on* Γ *, then*

(a)
Ψ(α,α)=∪{Ψ(β,γ)|β,γ∈Γ} *.(reflexive)*
(b)
Ψ(α,β)=Ψ(β,α) *.(symmetric)*
(c)
Ψ(α,γ)⊇Ψ(α,β)∩Ψ(β,γ) *.(transitive)*

Definition 5.4 *A hesitant fuzzy equivalence relation* Ψ *on* Γ *is called a hesitant fuzzy congruence relation on* Γ *if*

(a)
Ψ(α+γ,β+δ)⊇Ψ(α,β)∩Ψ(γ,δ)∀α,β,γ,δ∈Γ ,
(b)
Ψ(α⋆γ,β⋆δ)⊇Ψ(α,β)∩Ψ(γ,δ)∀α,β,γ,δ∈Γ ,
(c) For any α,β,γ,δ∈Γ , if γ⋆δ≼α⋆β , then Ψ(γ,δ)⊇Ψ(α,β) .

Example 5.5 *In* *Example 3.2* *,* Γ *becomes an autometrized algebra. Define a hesitant fuzzy relation* Ψ:Γ×Γ→P([0,1]) *on* Γ *by:* Ψ(0,0)=Ψ(α,α)=Ψ(β,β)=Ψ(γ,γ)={0.2,0.8} *and* Ψ(0,α)=Ψ(α,0)=Ψ(β,γ)=Ψ(γ,β)={0.8} *.*

*Now, check the three closure properties,*

(i)
$$\begin{array}{l} \Psi \left(\alpha +\beta ,\alpha +\beta \right)=\Psi \left(\gamma ,\gamma \right)=\left\{0.2,0.8\right\}⊇\Psi \left(\alpha ,\alpha \right)\cap \Psi \left(\beta ,\beta \right)=\left\{0.2,0.8\right\}\\ \Psi \left(\alpha +\gamma ,\alpha +\gamma \right)=\Psi \left(\gamma ,\gamma \right)=\left\{0.2,0.8\right\}⊇\Psi \left(\alpha ,\alpha \right)\cap \Psi \left(\gamma ,\gamma \right)=\left\{0.2,0.8\right\}\\ \Psi \left(\beta +\gamma ,\beta +\gamma \right)=\Psi \left(\gamma ,\gamma \right)=\left\{0.2,0.8\right\}⊇\Psi \left(\beta ,\beta \right)\cap \Psi \left(\gamma ,\gamma \right)=\left\{0.2,0.8\right\}\\ \Psi \left(\alpha +0,\alpha +\alpha \right)=\Psi \left(\alpha ,\alpha \right)=\left\{0.2,0.8\right\}⊇\Psi \left(\alpha ,\alpha \right)\cap \Psi \left(0,\alpha \right)=\left\{0.8\right\}\\ \Psi \left(\alpha +\beta ,\alpha +\gamma \right)=\Psi \left(\gamma ,\gamma \right)=\left\{0.2,0.8\right\}⊇\Psi \left(\alpha ,\alpha \right)\cap \Psi \left(\beta ,\gamma \right)=\left\{0.8\right\}\\ \Psi \left(\beta +0,\beta +\alpha \right)=\Psi \left(\beta ,\gamma \right)=\left\{0.8\right\}⊇\Psi \left(\beta ,\beta \right)\cap \Psi \left(0,\alpha \right)=\left\{0.8\right\}\\ \Psi \left(\beta +\beta ,\beta +\gamma \right)=\Psi \left(\beta ,\gamma \right)=\left\{0.8\right\}⊇\Psi \left(\beta ,\beta \right)\cap \Psi \left(\beta ,\gamma \right)=\left\{0.8\right\}\\ \Psi \left(\gamma +0,\gamma +\alpha \right)=\Psi \left(\gamma ,\gamma \right)=\left\{0.2,0.8\right\}⊇\Psi \left(\gamma ,\gamma \right)\cap \Psi \left(0,\alpha \right)=\left\{0.8\right\}\\ \Psi \left(\gamma +\beta ,\gamma +\gamma \right)=\Psi \left(\gamma ,\gamma \right)=\left\{0.2,0.8\right\}⊇\Psi \left(\gamma ,\gamma \right)\cap \Psi \left(\beta ,\gamma \right)=\left\{0.8\right\} \end{array}$$
(ii)
$$\begin{array}{l} \Psi \left(\alpha ⋆\beta ,\alpha ⋆\beta \right)=\Psi \left(\gamma ,\gamma \right)=\left\{0.2,0.8\right\}⊇\Psi \left(\alpha ,\alpha \right)\cap \Psi \left(\beta ,\beta \right)=\left\{0.2,0.8\right\}\\ \Psi \left(\alpha ⋆\gamma ,\alpha ⋆\gamma \right)=\Psi \left(\beta ,\beta \right)=\left\{0.2,0.8\right\}⊇\Psi \left(\alpha ,\alpha \right)\cap \Psi \left(\gamma ,\gamma \right)=\left\{0.2,0.8\right\}\\ \Psi \left(\beta ⋆\gamma ,\beta ⋆\gamma \right)=\Psi \left(\alpha ,\alpha \right)=\left\{0.2,0.8\right\}⊇\Psi \left(\beta ,\beta \right)\cap \Psi \left(\gamma ,\gamma \right)=\left\{0.2,0.8\right\}\\ \Psi \left(\alpha ⋆0,\alpha ⋆\alpha \right)=\Psi \left(\alpha ,0\right)=\left\{0.8\right\}⊇\Psi \left(\alpha ,\alpha \right)\cap \Psi \left(0,\alpha \right)=\left\{0.8\right\}\\ \Psi \left(\alpha ⋆\beta ,\alpha ⋆\gamma \right)=\Psi \left(\gamma ,\beta \right)=\left\{0.8\right\}⊇\Psi \left(\alpha ,\alpha \right)\cap \Psi \left(\beta ,\gamma \right)=\left\{0.8\right\}\\ \Psi \left(\beta ⋆0,\beta ⋆\alpha \right)=\Psi \left(\beta ,\gamma \right)=\left\{0.8\right\}⊇\Psi \left(\beta ,\beta \right)\cap \Psi \left(0,\alpha \right)=\left\{0.8\right\}\\ \Psi \left(\beta ⋆\beta ,\beta ⋆\gamma \right)=\Psi \left(0,\alpha \right)=\left\{0.8\right\}⊇\Psi \left(\beta ,\beta \right)\cap \Psi \left(\beta ,\gamma \right)=\left\{0.8\right\}\\ \Psi \left(\gamma ⋆0,\gamma ⋆\alpha \right)=\Psi \left(\gamma ,\beta \right)=\left\{0.8\right\}⊇\Psi \left(\gamma ,\gamma \right)\cap \Psi \left(0,\alpha \right)=\left\{0.8\right\}\\ \Psi \left(\gamma ⋆\beta ,\gamma ⋆\gamma \right)=\Psi \left(\alpha ,0\right)=\left\{0.8\right\}⊇\Psi \left(\gamma ,\gamma \right)\cap \Psi \left(\beta ,\gamma \right)=\left\{0.8\right\} \end{array}$$
(iii) Here, 0=0⋆0=α⋆α=β⋆β=γ⋆γ⇒Ψ(0,0)=Ψ(α,α)=Ψ(β,β)=Ψ(γ,γ)={0.2,0.8} and α=0⋆α=β⋆γ⇒Ψ(0,α)=Ψ(β,γ)={0.8}. Also, 0⋆0≼0⋆α⇒Ψ(0,0)={0.2,0.8}⊇Ψ(0,α)={0.8}

*
Therefore,* Ψ *is a hesitant fuzzy congruence relation on* Γ *.*

Definition 5.6 *If* Ψ *is a relation on* Γ *, then characteristic hesitant fuzzy relation* $\chi_{\Psi }$ *on* Γ *is a function of* Γ×Γ *into* P([0,1]) *defined as for all* (α,β)∈Γ×Γ *:*

$$\chi_{\Psi }\left(\alpha ,\;\beta \right)=\left\{ \begin{array}{ll} \left[0,\;1\right], & \text{if}\;\left(\alpha ,\;\beta \right)\in \Psi .\\ \emptyset , & \text{otherwise}. \end{array}\right.$$

Theorem 5.7 *A nonempty equivalent relation* Ψ *on* Γ *is a congruence relation on* Γ *if and only if the characteristic hesitant fuzzy equivalent relation* $\chi_{\Psi }$ *is a hesitant fuzzy congruence relation on* Γ *.*

Proof.
Assume that Ψ is a congruence relation on Γ . To show that $\chi_{\Psi }$ is a hesitant fuzzy congruence relation on Γ . Let α,β,γ,δ∈Γ .

(i) To show that $\chi_{\Psi }\left(\alpha +\gamma ,\;\beta +\delta \right)⊇\chi_{\Psi }\left(\alpha ,\;\beta \right)\cap \chi_{\Psi }\left(\gamma ,\;\delta \right)$ . Here we will consider two cases.
  (a) Let (α,β),(γ,δ)∈Ψ . Clearly, $\chi_{\Psi }\left(\alpha ,\;\beta \right)=\left[0,\;1\right]$ and $\chi_{\Psi }\left(\gamma ,\;\delta \right)=\left[0,\;1\right]$ . So, $\chi_{\Psi }\left(\alpha ,\;\beta \right)\cap \chi_{\Psi }\left(\gamma ,\;\delta \right)=\left[0,\;1\right]$ . Since Ψ is a congruence relation on Γ ; (α+γ,β+δ)∈Ψ . As a result, $\chi_{\Psi }\left(\alpha +\gamma ,\;\beta +\delta \right)=\left[0,\;1\right]$ . Therefore, $\chi_{\Psi }\left(\alpha +\gamma ,\;\beta +\delta \right)⊇\chi_{\Psi }\left(\alpha ,\;\beta \right)\cap \chi_{\Psi }\left(\gamma ,\;\delta \right)$ .
  (b) Let (α,β)∉Ψ or (γ,δ)∉Ψ . Clearly, $\chi_{\Psi }\left(\alpha ,\;\beta \right)=\emptyset$ or $\chi_{\Psi }\left(\gamma ,\;\delta \right)=\emptyset$ . So, $\chi_{\Psi }\left(\alpha ,\;\beta \right)\cap \chi_{\Psi }\left(\gamma ,\;\delta \right)=\emptyset$ . Therefore, $\chi_{\Psi }\left(\alpha +\gamma ,\;\beta +\delta \right)⊇\chi_{\Psi }\left(\alpha ,\;\beta \right)\cap \chi_{\Psi }\left(\gamma ,\;\delta \right)$ .
(ii) To show that $\chi_{\Psi }\left(\alpha ⋆\gamma ,\;\beta ⋆\delta \right)⊇\chi_{\Psi }\left(\alpha ,\;\beta \right)\cap \chi_{\Psi }\left(\gamma ,\;\delta \right)$ . Here we will consider two cases.
  (a) Let (α,β),(γ,δ)∈Ψ . Clearly, $\chi_{\Psi }\left(\alpha ,\;\beta \right)=\left[0,\;1\right]$ and $\chi_{\Psi }\left(\gamma ,\;\delta \right)=\left[0,\;1\right]$ . So, $\chi_{\Psi }\left(\alpha ,\;\beta \right)\cap \chi_{\Psi }\left(\gamma ,\;\delta \right)=\left[0,\;1\right]$ . Since Ψ is a congruence relation on Γ ; (α⋆γ,β⋆δ)∈Ψ . As a result, $\chi_{\Psi }\left(\alpha ⋆\gamma ,\;\beta ⋆\delta \right)=\left[0,\;1\right]$ . Therefore, $\chi_{\Psi }\left(\alpha ⋆\gamma ,\;\beta ⋆\delta \right)⊇\chi_{\Psi }\left(\alpha ,\;\beta \right)\cap \chi_{\Psi }\left(\gamma ,\;\delta \right)$ .
  (b) Let (α,β)∉Ψ or (γ,δ)∉Ψ . Clearly, $\chi_{\Psi }\left(\alpha ,\;\beta \right)=\emptyset$ or $\chi_{\Psi }\left(\gamma ,\;\delta \right)=\emptyset$ . So, $\chi_{\Psi }\left(\alpha ,\;\beta \right)\cap \chi_{\Psi }\left(\gamma ,\;\delta \right)=\emptyset$ . Therefore, $\chi_{\Psi }\left(\alpha ⋆\gamma ,\;\beta ⋆\delta \right)⊇\chi_{\Psi }\left(\alpha ,\;\beta \right)\cap \chi_{\Psi }\left(\gamma ,\;\delta \right)$ .
(iii) Let α,β,γ,δ∈Γ and suppose that γ⋆δ≼α⋆β . To show that $\chi_{\Psi }\left(\gamma ,\;\delta \right)⊇\chi_{\Psi }\left(\alpha ,\;\beta \right)$ . Here we will consider two cases.
  (a) Let (α,β)∈Ψ . Clearly, $\chi_{\Psi }\left(\alpha ,\;\beta \right)=\left[0,\;1\right]$ . Since Ψ is a congruence relation on Γ ; (γ,δ)∈Ψ . As a result, $\chi_{\Psi }\left(\gamma ,\;\delta \right)=\left[0,\;1\right]$ . Therefore, $\chi_{\Psi }\left(\gamma ,\;\delta \right)⊇\chi_{\Psi }\left(\alpha ,\;\beta \right)$ .
  (b) Let (α,β)∉Ψ . Clearly, $\chi_{\Psi }\left(\alpha ,\;\beta \right)=\emptyset$ . Therefore, $\chi_{\Psi }\left(\gamma ,\;\delta \right)⊇\chi_{\Psi }\left(\alpha ,\;\beta \right)$ . Hence, $\chi_{\Psi }$ is a hesitant fuzzy congruence relation on Γ .

Conversely, assume that $\chi_{\Psi }$ is a hesitant fuzzy congruence relation on Γ . To show that Ψ is a congruence relation on Γ .

(i) Let (α,β),(γ,δ)∈Ψ . Then, $\chi_{\Psi }\left(\alpha ,\;\beta \right)=\left[0,\;1\right]$ and $\chi_{\Psi }\left(\gamma ,\;\delta \right)=\left[0,\;1\right]$ . Since $\chi_{\Psi }$ is a hesitant fuzzy congruence relation on Γ ; $\chi_{\Psi }\left(\alpha +\gamma ,\;\beta +\delta \right)⊇\chi_{\Psi }\left(\alpha ,\;\beta \right)\cap \chi_{\Psi }\left(\gamma ,\;\delta \right)=\left[0,\;1\right]$ . So, $\chi_{\Psi }\left(\alpha +\gamma ,\;\beta +\delta \right)=\left[0,\;1\right]$ . Hence, (α+γ,β+δ)∈Ψ .
(ii) Let (α,β),(γ,δ)∈Ψ . Then, $\chi_{\Psi }\left(\alpha ,\;\beta \right)=\left[0,\;1\right]$ and $\chi_{\Psi }\left(\gamma ,\;\delta \right)=\left[0,\;1\right]$ . Since $\chi_{\Psi }$ is a hesitant fuzzy congruence relation on Γ ; $\chi_{\Psi }\left(\alpha ⋆\gamma ,\;\beta ⋆\delta \right)⊇\chi_{\Psi }\left(\alpha ,\;\beta \right)\cap \chi_{\Psi }\left(\gamma ,\;\delta \right)=\left[0,\;1\right]$ . So, $\chi_{\Psi }\left(\alpha ⋆\gamma ,\;\beta ⋆\delta \right)=\left[0,\;1\right]$ . Hence, (α⋆γ,β⋆δ)∈Ψ .
(iii) Let (α,β)∈Ψ and γ⋆δ≼α⋆β . Clearly, $\chi_{\Psi }\left(\alpha ,\;\beta \right)=\left[0,\;1\right]$ . Since Ψ is a hesitant fuzzy congruence relation on Γ ; then $\chi_{\Psi }\left(\gamma ,\;\delta \right)⊇\chi_{\Psi }\left(\alpha ,\;\beta \right)=\left[0,\;1\right]$ . Therefore, $\chi_{\Psi }\left(\gamma ,\;\delta \right)=\left[0,\;1\right]$ . Hence, (γ,δ)∈Ψ . Hence, Ψ is a congruence relation on Γ .

Let Ψ be a hesitant fuzzy relation on Γ . For all ε∈P([0,1]) , the sets $F^{⊇}\left(\Psi ,\;\varepsilon \right)=\left\{\left(\alpha ,\;\beta \right)\in \Gamma \times \Gamma |\Psi \left(\alpha ,\;\beta \right)⊇\varepsilon \right\}$ , $F^{⊃}\left(\Psi ,\;\varepsilon \right)=\left\{\left(\alpha ,\;\beta \right)\in \Gamma \times \Gamma |\Psi \left(\alpha ,\;\beta \right)⊃\varepsilon \right\}$ , $F^{\subseteq }\left(\Psi ,\;\varepsilon \right)=\left\{\left(\alpha ,\;\beta \right)\in \Gamma \times \Gamma |\Psi \left(\alpha ,\;\beta \right)\subseteq \varepsilon \right\}$ , and $F^{\subset }\left(\Psi ,\;\varepsilon \right)=\left\{\left(\alpha ,\;\beta \right)\in \Gamma \times \Gamma |\Psi \left(\alpha ,\;\beta \right)\subset \varepsilon \right\}$ are called level subset of Ψ .

Theorem 5.8 *Let* Ψ *be a hesitant fuzzy relation on* Γ *.* Ψ *is a hesitant fuzzy congruence relation on* Γ *if and only if for all* t∈P([0,1]) *,* $F^{⊇}\left(\Psi ,\;\varepsilon \right)$ *is either empty or a congruence relation on* Γ *.*

Proof.
Assume that Ψ is a hesitant fuzzy congruence relation on Γ .

(i) Since $F^{⊇}\left(\Psi ,\;\varepsilon \right)$ is a nonempty; let $\left(\alpha ,\;\beta \right)\in F^{⊇}\left(\Psi ,\;\varepsilon \right)$ . So, Ψ(α,β)⊇ε . But Ψ(α,α)=∪{Ψ(β,γ)|β,γ∈Γ}⊇Ψ(α,β)⊇ε . So, Ψ(α,α)⊇ε . Therefore, $\left(\alpha ,\;\alpha \right)\in F^{⊇}\left(\Psi ,\;\varepsilon \right)$ .
(ii) Let $\left(\alpha ,\;\beta \right)\in F^{⊇}\left(\Psi ,\;\varepsilon \right)$ . So, Ψ(α,β)⊇ε . Clearly, Ψ(α,β)=Ψ(β,α)⊇ε . So, Ψ(β,α)⊇ε . Therefore, $\left(\beta ,\;\alpha \right)\in F^{⊇}\left(\Psi ,\;\varepsilon \right)$ .
(iii) Let $\left(\alpha ,\;\beta \right),\;\left(\beta ,\;\gamma \right)\in F^{⊇}\left(\Psi ,\;\varepsilon \right)$ . Then, Ψ(α,β)⊇ε and Ψ(β,γ)⊇ε . Clearly, Ψ(α,β)∩Ψ(β,γ)⊇ε . Since Ψ is a hesitant fuzzy congruence; Ψ(α,γ)⊇Ψ(α,β)∩Ψ(β,γ)⊇ε . Therefore, $\left(\alpha ,\;\gamma \right)\in F^{⊇}\left(\Psi ,\;\varepsilon \right)$ . Therefore, $F^{⊇}\left(\Psi ,\;\varepsilon \right)$ is an equivalence relation.
  (a) Let $\left(\alpha ,\;\beta \right),\;\left(\gamma ,\;\delta \right)\in F^{⊇}\left(\Psi ,\;\varepsilon \right)$ . So, Ψ(α,β)⊇ε and Ψ(γ,δ)⊇ε . Since Ψ is a hesitant fuzzy congruence relation on Γ ; Ψ(α+γ,β+δ)⊇Ψ(α,β)∩Ψ(γ,δ)⊇ε . Therefore, $\left(\alpha +\gamma ,\;\beta +\delta \right)\in F^{⊇}\left(\Psi ,\;\varepsilon \right)$ .
  (b) Let $\left(\alpha ,\;\beta \right),\;\left(\gamma ,\;\delta \right)\in F^{⊇}\left(\Psi ,\;\varepsilon \right)$ . So, Ψ(α,β)⊇ε and Ψ(γ,δ)⊇ε . Since Ψ is a hesitant fuzzy congruence relation on Γ ; Ψ(α⋆γ,β⋆δ)⊇Ψ(α,β)∩Ψ(γ,δ)⊇ε . Therefore, $\left(\alpha ⋆\gamma ,\;\beta ⋆\delta \right)\in F^{⊇}\left(\Psi ,\;\varepsilon \right)$ .
  (c) Let $\left(\alpha ,\;\beta \right)\in F^{⊇}\left(\Psi ,\;\varepsilon \right)$ and γ⋆δ≼α⋆β . Clearly, (α,β)⊇ε . Since Ψ is a hesitant fuzzy congruence relation on Γ ; then Ψ(γ,δ)⊇Ψ(α,β)⊇ε . Therefore, $\left(\gamma ,\;\delta \right)\in F^{⊇}\left(\Psi ,\;\varepsilon \right)$ . Hence, $F^{⊇}\left(\Psi ,\;\varepsilon \right)$ is a congruence relation on Γ .
  Conversely, $F^{⊇}\left(\Psi ,\;\varepsilon \right)$ is a congruence relation on Γ . To show that Ψ is a hesitant fuzzy congruence relation on Γ .
(i) We know that for any α,β,γ∈Γ , γ⋆γ=0≼α⋆β . Take ε=Ψ(α,β) . So, $\left(\alpha ,\;\beta \right)\in F^{⊇}\left(\Psi ,\;\varepsilon \right)$ . Since $F^{⊇}\left(\Psi ,\;\varepsilon \right)$ is a congruence relation; $\left(\gamma ,\;\gamma \right)\in F^{⊇}\left(\Psi ,\;\varepsilon \right)$ . Then, Ψ(γ,γ)⊇ε=Ψ(α,β) . Therefore, Ψ(γ,γ)=∪{Ψ(α,β)|α,β∈Γ} .
(ii) Let α,β∈Γ . Take ε=Ψ(α,β) . So, $\left(\alpha ,\;\beta \right)\in F^{⊇}\left(\Psi ,\;\varepsilon \right)$ . Since $F^{⊇}\left(\Psi ,\;\varepsilon \right)$ is a congruence; $\left(\beta ,\;\alpha \right)\in F^{⊇}\left(\Psi ,\;\varepsilon \right)$ . So, Ψ(β,α)⊇ε . Clearly, Ψ(β,α)⊇Ψ(α,β) . Again, take ε=Ψ(β,α) . So, $\left(\beta ,\;\alpha \right)\in F^{⊇}\left(\Psi ,\;\varepsilon \right)$ . Since $F^{⊇}\left(\Psi ,\;\varepsilon \right)$ is a congruence; $\left(\alpha ,\;\beta \right)\in F^{⊇}\left(\Psi ,\;\varepsilon \right)$ . So, Ψ(α,β)⊇ε . Clearly, Ψ(α,β)⊇Ψ(β,α) . Consequently, Ψ(α,β)=Ψ(β,α) .
(iii) Let α,β,γ∈Γ . Take ε=Ψ(α,β)∩Ψ(β,γ) . Clearly, Ψ(α,β)⊇ε and Ψ(β,γ)⊇ε . This implies that $\left(\alpha ,\;\beta \right),\;\left(\beta ,\;\gamma \right)\in F^{⊇}\left(\Psi ,\;\varepsilon \right)$ . Since $F^{⊇}\left(\Psi ,\;\varepsilon \right)$ is a congruence relation; $\left(\alpha ,\;\gamma \right)\in F^{⊇}\left(\Psi ,\;\varepsilon \right)$ . Therefore, Ψ(α,γ)⊇ε . Thus, Ψ(α,γ)⊇Ψ(α,β)∩Ψ(β,γ) . Therefore, Ψ is a hesitant equivalence relation.
  (a) Let α,β,γ,δ∈Γ . Take ε=Ψ(α,β)∩Ψ(γ,δ) . Therefore, $\left(\alpha ,\;\beta \right)\in F^{⊇}\left(\Psi ,\;\varepsilon \right)$ and $\left(\gamma ,\;\delta \right)\in F^{⊇}\left(\Psi ,\;\varepsilon \right)$ . Since $F^{⊇}\left(\Psi ,\;\varepsilon \right)$ is a congruence relation on Γ ; $\left(\alpha +\gamma ,\;\beta +\delta \right)\in F^{⊇}\left(\Psi ,\;\varepsilon \right)$ . As a result, (α+γ,β+δ)⊇ε=Ψ(α,β)∩Ψ(γ,δ) .
  (b) Let α,β,γ,δ∈Γ . Take ε=Ψ(α,β)∩Ψ(γ,δ) . Therefore, $\left(\alpha ,\;\beta \right)\in F^{⊇}\left(\Psi ,\;\varepsilon \right)$ and $\left(\gamma ,\;\delta \right)\in F^{⊇}\left(\Psi ,\;\varepsilon \right)$ . Since $F^{⊇}\left(\Psi ,\;\varepsilon \right)$ is a congruence relation on Γ ; $\left(\alpha ⋆\gamma ,\;\beta ⋆\delta \right)\in F^{⊇}\left(\Psi ,\;\varepsilon \right)$ . As a result, (α⋆γ,β⋆δ)⊇ε=Ψ(α,β)∩Ψ(γ,δ) .
  (c) Let α,β,γ,δ∈Γ and suppose that γ⋆δ≼α⋆β . Take ε=Ψ(α,β) . Therefore, $\left(\alpha ,\;\beta \right)\in F^{⊇}\left(\Psi ,\;\varepsilon \right)$ . Since $F^{⊇}\left(\Psi ,\;\varepsilon \right)$ is a congruence relation on Γ ; $\left(\gamma ,\;\delta \right)\in F^{⊇}\left(\Psi ,\;\varepsilon \right)$ . Therefore, Ψ(γ,δ)⊇ε=Ψ(α,β) . Hence, Ψ is a hesitant fuzzy congruence relation on Γ .

Theorem 5.9 *Let* Ψ *be a hesitant fuzzy equivalent relation on* Γ *. If* Im(Ψ) *is a chain and for all* ε∈P([0,1]) *, a nonempty subset* $F^{⊃}\left(\Psi ,\;\varepsilon \right)$ *is a congruence relation on* Γ *, then* Ψ *is a hesitant fuzzy congruence relation on* Γ *.*

Proof.
Assume that Im(Ψ) is a chain and for all ε∈P([0,1]) , a nonempty subset $F^{⊃}\left(\Psi ,\;\varepsilon \right)$ is a congruence relation on Γ . To show that Ψ is a hesitant fuzzy congruence relation on Γ .

(i) Let α,β,γ,δ∈Γ . To show that Ψ(α+γ,β+δ)⊇Ψ(α,β)∩Ψ(γ,δ) . Suppose that Ψ(α+γ,β+δ)⊉Ψ(α,β)∩Ψ(γ,δ) . Since Im(Ψ) is a chain; implies that Ψ(α+γ,β+δ)⊂Ψ(α,β)∩Ψ(γ,δ) . Then, Ψ(α+γ,β+δ)∈P([0,1]) . Take ε=Ψ(α+γ,β+δ) . Therefore, Ψ(α,β)⊃ε and Ψ(γ,δ)⊃ε . As a result, $\left(\alpha ,\;\beta \right),\;\left(\gamma ,\;\delta \right)\in F^{⊃}\left(\Psi ,\;\varepsilon \right)\neq \emptyset$ . Since $F^{⊃}\left(\Psi ,\;\varepsilon \right)$ is a congruence relation on Γ ; $\left(\alpha +\gamma ,\;\beta +\delta \right)\in F^{⊃}\left(\Psi ,\;\varepsilon \right)$ . So, Ψ(α+γ,β+δ)⊃ε=Ψ(α+γ,β+δ) . This is a contradiction. Thus, Ψ(α+γ,β+δ)⊇Ψ(α,β)∩Ψ(γ,δ) for all α,β,γ,δ∈Γ .
(ii) Let α,β,γ,δ∈Γ . To show that Ψ(α⋆γ,β⋆δ)⊇Ψ(α,β)∩Ψ(γ,δ) . Suppose that Ψ(α⋆γ,β⋆δ)⊉Ψ(α,β)∩Ψ(γ,δ) . Since Im(Ψ) is a chain; implies that Ψ(α⋆γ,β⋆δ)⊂Ψ(α,β)∩Ψ(γ,δ) . Then, Ψ(α⋆γ,β⋆δ)∈P([0,1]) . Take ε=Ψ(α⋆γ,β⋆δ) . Therefore, Ψ(α,β)⊃ε and Ψ(γ,δ)⊃ε . As a result, $\left(\alpha ,\;\beta \right),\;\left(\gamma ,\;\delta \right)\in F^{⊃}\left(\Psi ,\;\varepsilon \right)\neq \emptyset$ . Since $F^{⊃}\left(\Psi ,\;\varepsilon \right)$ is a congruence relation on Γ ; $\left(\alpha ⋆\gamma ,\;\beta ⋆\delta \right)\in F^{⊃}\left(\Psi ,\;\varepsilon \right)$ . So, Ψ(α⋆γ,β⋆δ)⊃ε=Ψ(α⋆γ,β⋆δ) . This is a contradiction. Thus, Ψ(α⋆γ,β⋆δ)⊇Ψ(α,β)∩Ψ(γ,δ) for all α,β,γ,δ∈Γ .
(iii) Let α,β,γ,δ∈Γ and suppose that γ⋆δ≼α⋆β . To show that Ψ(γ,δ)⊇Ψ(α,β) . Suppose that Suppose that Ψ(γ,δ)⊉Ψ(α,β) . Since Im(Ψ) is a chain; implies that Ψ(γ,δ)⊂Ψ(α,β) . Take ε=Ψ(γ,δ) . Therefore, $\left(\alpha ,\;\beta \right)\in F^{⊃}\left(\Psi ,\;\varepsilon \right)$ . Since $F^{⊃}\left(\Psi ,\;\varepsilon \right)$ is a congruence relation on relation on Γ ; $\left(\gamma ,\;\delta \right)\in F^{⊃}\left(\Psi ,\;\varepsilon \right)$ . Therefore, Ψ(γ,δ)⊃ε=Ψ(γ,δ) is contradiction. Therefore, Ψ(γ,δ)⊇Ψ(α,β) . Hence, Ψ is a hesitant fuzzy congruence relation on Γ .

Theorem 5.10 *Let* $\bar{\Psi }$ *be a hesitant fuzzy equivalent relation on* Γ *. Then* $\bar{\Psi }$ *is a hesitant fuzzy congruence relation on* Γ *if and only if for all* ε∈P([0,1]) *,* $F^{\subseteq }\left(\Psi ,\;\varepsilon \right)$ *is either empty or congruence relation on* Γ *.*

Proof.
Assume that $\bar{\Psi }$ is a hesitant fuzzy congruence relation on Γ . To show that $F^{\subseteq }\left(\Psi ,\;\varepsilon \right)$ is a congruence relation on Γ . Let ε∈P([0,1]) be such that $F^{\subseteq }\left(\Psi ,\;\varepsilon \right)\neq \emptyset$ .

(i) Since $F^{\subseteq }\left(\Psi ,\;\varepsilon \right)$ is a nonempty; let $\left(\alpha ,\;\beta \right)\in F^{\subseteq }\left(\Psi ,\;\varepsilon \right)$ . So, Ψ(α,β)⊆ε . Since $\bar{\Psi }$ is a hesitant fuzzy congruence relation on Γ ; $\bar{\Psi }\left(\alpha ,\;\alpha \right)=\cup \left\{\bar{\Psi }\left(\beta ,\;\gamma \right)|\beta ,\;\gamma \in \Gamma \right\}$ . Then, [0,1]\Ψ(α,α)=[0,1]\∩{Ψ(β,γ)|β,γ∈Γ} . As a result, Ψ(α,α)=∩{Ψ(β,γ)|β,γ∈Γ}⊆Ψ(α,β)⊆ε . Therefore, $\left(\alpha ,\;\alpha \right)\in F^{\subseteq }\left(\Psi ,\;\varepsilon \right)$ .
(ii) Let $\left(\alpha ,\;\beta \right)\in F^{\subseteq }\left(\Psi ,\;\varepsilon \right)$ . So, Ψ(α,β)⊆ε . Since $\bar{\Psi }$ is a hesitant fuzzy congruence relation on Γ ; $\bar{\Psi }\left(\alpha ,\;\beta \right)=\bar{\Psi }\left(\beta ,\;\alpha \right)$ . Clearly, Ψ(α,β)=Ψ(β,α)⊆ε . So, Ψ(β,α)⊆ε . Therefore, $\left(\beta ,\;\alpha \right)\in F^{\subseteq }\left(\Psi ,\;\varepsilon \right)$ .
(iii) Let $\left(\alpha ,\;\beta \right),\;\left(\beta ,\;\gamma \right)\in F^{\subseteq }\left(\Psi ,\;\varepsilon \right)$ . Then, Ψ(α,β)⊆ε and Ψ(β,γ)⊆ε . Since $\bar{\Psi }$ is a hesitant fuzzy congruence relation on Γ ; $\bar{\Psi }\left(\alpha ,\;\gamma \right)⊇\bar{\Psi }\left(\alpha ,\;\beta \right)\cap \bar{\Psi }\left(\beta ,\;\gamma \right)$ . Then, [0,1]\Ψ(α,γ)⊇([0,1]\Ψ(α,β))∩([0,1]\Ψ(β,γ))=[0,1]\Ψ(α,β)∪Ψ(β,γ) . Consequently, Ψ(α,γ)⊆Ψ(α,β)∪Ψ(β,γ)⊆ε . Therefore, $\left(\alpha ,\;\gamma \right)\in F^{\subseteq }\left(\Psi ,\;\varepsilon \right)$ . Therefore, $F^{\subseteq }\left(\Psi ,\;\varepsilon \right)$ is an equivalence relation.
  (a) Let $\left(\alpha ,\;\beta \right),\;\left(\gamma ,\;\delta \right)\in F^{\subseteq }\left(\Psi ,\;\varepsilon \right)$ . Then, Ψ(α,β)⊆ε and Ψ(γ,δ)⊆ε . Since $\bar{\Psi }$ is a hesitant fuzzy congruence relation on Γ ; $\bar{\Psi }\left(\alpha +\gamma ,\;\beta +\delta \right)⊇\bar{\Psi }\left(\alpha ,\;\beta \right)\cap \bar{\Psi }\left(\gamma ,\;\delta \right)$ . So, [0,1]\Ψ(α+γ,β+δ)⊇([0,1]\Ψ(α,β))∩([0,1]\Ψ(γ,δ))=([0,1]\(Ψ(α,β)∪Ψ(γ,δ)) . Therefore, Ψ(α+γ,β+δ)⊆Ψ(α,β)∪Ψ(γ,δ)⊆ε . Hence, $\left(\alpha +\gamma ,\;\beta +\delta \right)\in F^{\subseteq }\left(\Psi ,\;\varepsilon \right)$ .
  (b) Let $\left(\alpha ,\;\beta \right),\;\left(\gamma ,\;\delta \right)\in F^{\subseteq }\left(\Psi ,\;\varepsilon \right)$ . Then, Ψ(α,β)⊆ε and Ψ(γ,δ)⊆ε . Since $\bar{\Psi }$ is a hesitant fuzzy congruence relation on Γ ; $\bar{\Psi }\left(\alpha ⋆\gamma ,\;\beta ⋆\delta \right)⊇\bar{\Psi }\left(\alpha ,\;\beta \right)\cap \bar{\Psi }\left(\gamma ,\;\delta \right)$ . So, [0,1]\Ψ(α⋆γ,β⋆δ)⊇([0,1]\Ψ(α,β))∩([0,1]\Ψ(γ,δ))=([0,1]\(Ψ(α,β)∪Ψ(γ,δ)) . Therefore, Ψ(α⋆γ,β⋆δ)⊆Ψ(α,β)∪Ψ(γ,δ)⊆ε . Hence, $\left(\alpha ⋆\gamma ,\;\beta ⋆\delta \right)\in F^{\subseteq }\left(\Psi ,\;\varepsilon \right)$ .
  (c) Let $\left(\alpha ,\;\beta \right)\in F^{\subseteq }\left(\Psi ,\;\varepsilon \right)$ and γ⋆δ≼α⋆β . Clearly, Ψ(α,β)⊆ε . To show that $\left(\gamma ,\;\delta \right)\in \delta )\in F^{\subseteq }\left(\Psi ,\;\varepsilon \right)$ . Since $\bar{\Psi }$ is a hesitant fuzzy congruence relation on Γ , $\bar{\Psi }\left(\gamma ,\;\delta \right)⊇\bar{\Psi }\left(\alpha ,\;\beta \right)$ . So, [0,1]\Ψ(γ,δ)⊇[0,1]\Ψ(α,β) . Therefore, Ψ(γ,δ)⊆Ψ(α,β)⊆ε . Thus, $\left(\gamma ,\;\delta \right)\in F^{\subseteq }\left(\Psi ,\;\varepsilon \right)$ . Hence, $F^{\subseteq }\left(\Psi ,\;\varepsilon \right)$ is congruence relation on Γ .

Conversely, assume that $F^{\subseteq }\left(\Psi ,\;\varepsilon \right)$ is congruence relation on Γ . To show that $\bar{\Psi }$ is a hesitant fuzzy congruence relation on Γ .

(i) We know that for any α,β,γ∈Γ , α⋆α=0≼β⋆γ . Take ε=Ψ(β,γ) . So, $\left(\beta ,\;\gamma \right)\in F^{\subseteq }\left(\Psi ,\;\varepsilon \right)$ . Since $F^{\subseteq }\left(\Psi ,\;\varepsilon \right)$ is a congruence relation; $\left(\alpha ,\;\alpha \right)\in F^{\subseteq }\left(\Psi ,\;\varepsilon \right)$ . Then, Ψ(α,α)⊆ε=Ψ(β,γ) . So, [0,1]\Ψ(α,α)⊇[0,1]\Ψ(β,γ) . Clearly, $\bar{\Psi }\left(\alpha ,\;\alpha \right)⊇\bar{\Psi }\left(\beta ,\;\gamma \right)$ . Therefore, $\bar{\Psi }\left(\alpha ,\;\alpha \right)=\cup \left\{\bar{\Psi }\left(\beta ,\;\gamma \right)|\beta ,\;\gamma \in \Gamma \right\}$ .
(ii) Let α,β∈Γ . Take ε=Ψ(α,β) . So, $\left(\alpha ,\;\beta \right)\in F^{\subseteq }\left(\Psi ,\;\varepsilon \right)$ . Since $F^{\subseteq }\left(\Psi ,\;\varepsilon \right)$ is a congruence; $\left(\beta ,\;\alpha \right)\in F^{\subseteq }\left(\Psi ,\;\varepsilon \right)$ . So, Ψ(β,α)⊆ε . Therefore, Ψ(β,α)⊆Ψ(α,β) . Clearly, [0,1]\Ψ(β,α)⊇[0,1]\Ψ(α,β) . Therefore, $\bar{\Psi }\left(\beta ,\;\alpha \right)⊇\bar{\Psi }\left(\alpha ,\;\beta \right)$ . Again, take ε=Ψ(β,α) . So, $\left(\beta ,\;\alpha \right)\in F^{\subseteq }\left(\Psi ,\;\varepsilon \right)$ . Since $F^{\subseteq }\left(\Psi ,\;\varepsilon \right)$ is a congruence; $\left(\alpha ,\;\beta \right)\in F^{\subseteq }\left(\Psi ,\;\varepsilon \right)$ . So, Ψ(α,β)⊆ε . Clearly, Ψ(α,β)⊆Ψ(β,α) . Clearly, [0,1]\Ψ(α,β)⊇[0,1]\Ψ(β,α) . Therefore, $\bar{\Psi }\left(\alpha ,\;\beta \right)⊇\bar{\Psi }\left(\beta ,\;\alpha \right)$ . Hence, $\bar{\Psi }\left(\alpha ,\;\beta \right)=\bar{\Psi }\left(\beta ,\;\alpha \right)$ .
(iii) Let α,β,γ∈Γ . Take ε=Ψ(α,β)∪Ψ(β,γ) . Clearly, Ψ(α,β)⊆ε and Ψ(β,γ)⊆ε . This implies that $\left(\alpha ,\;\beta \right),\;\left(\beta ,\;\gamma \right)\in F^{\subseteq }\left(\Psi ,\;\varepsilon \right)$ . Since $F^{\subseteq }\left(\Psi ,\;\varepsilon \right)$ is a congruence relation; $\left(\alpha ,\;\gamma \right)\in F^{\subseteq }\left(\Psi ,\;\varepsilon \right)$ . Therefore, Ψ(α,γ)⊆ε . Clearly, Ψ(α,γ)⊆Ψ(α,β)∪Ψ(β,γ) . Now, consider [0,1]\Ψ(α,γ)⊇([0,1]\Ψ(α,β)∪Ψ(β,γ))=([0,1]\Ψ(α,β))∩([0,1]\Ψ(β,γ)) . As a result, $\bar{\Psi }\left(\alpha ,\;\gamma \right)⊇\bar{\Psi }\left(\alpha ,\;\beta \right)\cap \bar{\Psi }\left(\beta ,\;\gamma \right)$ . Therefore, $\bar{\Psi }$ is a hesitant equivalence relation.
  (a) Let α,β,γ,δ∈Γ . Take ε=Ψ(α,β)∪Ψ(γ,δ) . Therefore, Ψ(α,β)⊆ε and Ψ(γ,δ)⊆ε . As a result, $\left(\alpha ,\;\beta \right),\;\left(\gamma ,\;\delta \right)\in F^{\subseteq }\left(\Psi ,\;\varepsilon \right)\neq \emptyset$ . Since $F^{\subseteq }\left(\Psi ,\;\varepsilon \right)$ is a congruence relation on Γ ; $\left(\alpha +\gamma ,\;\beta +\delta \right)\in F^{\subseteq }\left(\Psi ,\;\varepsilon \right)$ . Clearly, Ψ(α+γ,β+δ)⊆ε=Ψ(α,β)∪Ψ(γ,δ) . As a result, $\left[0,\;1\right]\backslash \Psi \left(\alpha +\gamma ,\;\beta +\delta \right)⊇\left[0,\;1\right]\backslash \left(\Psi \left(\alpha ,\;\beta \right)\cup \Psi \left(\gamma ,\;\delta \right)\right)=\left(\left[0,\;1\right]\backslash \Psi \left(\alpha ,\;\beta \right)\right)\cap \left(\left[0,\;1\right]\backslash \Psi \left(\gamma ,\;\delta \right)\right)=\bar{\Psi }\left(\alpha ,\;\beta \right)\cap \bar{\Psi }\left(\gamma ,\;\delta \right)$ . Therefore, $\bar{\Psi }\left(\alpha +\gamma ,\;\beta +\delta \right)⊇\bar{\Psi }\left(\alpha ,\;\beta \right)\cap \bar{\Psi }\left(\gamma ,\;\delta \right)$ .
  (b) Let α,β,γ,δ∈Γ . Take ε=Ψ(α,β)∪Ψ(γ,δ) . Therefore, Ψ(α,β)⊆ε and Ψ(γ,δ)⊆ε . As a result, $\left(\alpha ,\;\beta \right),\;\left(\gamma ,\;\delta \right)\in F^{\subseteq }\left(\Psi ,\;\varepsilon \right)\neq \emptyset$ . Since $F^{\subseteq }\left(\Psi ,\;\varepsilon \right)$ is a congruence relation on Γ ; $\left(\alpha ⋆\gamma ,\;\beta ⋆\delta \right)\in F^{\subseteq }\left(\Psi ,\;\varepsilon \right)$ . Clearly, Ψ(α⋆γ,β⋆δ)⊆ε=Ψ(α,β)∪Ψ(γ,δ) . As a result, $\left[0,\;1\right]\backslash \Psi \left(\alpha ⋆\gamma ,\;\beta ⋆\delta \right)⊇\left[0,\;1\right]\backslash \left(\Psi \left(\alpha ,\;\beta \right)\cup \Psi \left(\gamma ,\;\delta \right)\right)=\left(\left[0,\;1\right]\backslash \Psi \left(\alpha ,\;\beta \right)\right)\cap \left(\left[0,\;1\right]\backslash \Psi \left(\gamma ,\;\delta \right)\right)=\bar{\Psi }\left(\alpha ,\;\beta \right)\cap \bar{\Psi }\left(\gamma ,\;\delta \right)$ . Therefore, $\bar{\Psi }\left(\alpha ⋆\gamma ,\;\beta ⋆\delta \right)⊇\bar{\Psi }\left(\alpha ,\;\beta \right)\cap \bar{\Psi }\left(\gamma ,\;\delta \right)$ .
  (c) Let α,β,γ,δ∈Γ and suppose that γ⋆δ≼α⋆β . Take ε=Ψ(α,β) . So, $\left(\alpha ,\;\beta \right)\in F^{\subseteq }\left(\Psi ,\;\varepsilon \right)$ . Since $F^{\subseteq }\left(\Psi ,\;\varepsilon \right)$ is a congruence relation on Γ ; $\left(\gamma ,\;\delta \right)\in F^{\subseteq }\left(\Psi ,\;\varepsilon \right)$ . As a result, Ψ(γ,δ)⊆ε=Ψ(α,β) . Clearly, [0,1]\Ψ(γ,δ)⊇[0,1]\Ψ(α,β) . Therefore, $\bar{\Psi }\left(\gamma ,\;\delta \right)⊇\bar{\Psi }\left(\alpha ,\;\beta \right)$ . Hence, $\bar{\Psi }$ is a hesitant fuzzy congruence relation on Γ .

Theorem 5.11 *Let* Ψ *be a hesitant fuzzy equivalent relation on* Γ *. If* Im(Ψ) *is a chain and for all* ε∈P([0,1]) *, a nonempty subset* $F^{\subset }\left(\Psi ,\;\varepsilon \right)$ *is a congruence relation on* Γ *, then* $\bar{\Psi }$ *is a hesitant fuzzy congruence relation on* Γ *.*

Proof.
Assume that Im(Ψ) is a chain and for all ε∈P([0,1]) , a nonempty subset $F^{\subset }\left(\Psi ,\;\varepsilon \right)$ is a congruence relation on Γ . To show that $\bar{\Psi }$ is a hesitant fuzzy congruence relation on Γ .

(i) Let α,β,γ,δ∈Γ . To show that $\bar{\Psi }\left(\alpha +\gamma ,\;\beta +\delta \right)⊇\bar{\Psi }\left(\alpha ,\;\beta \right)\cap \bar{\Psi }\left(\gamma ,\;\delta \right)$ . Suppose that $\bar{\Psi }\left(\alpha +\gamma ,\;\beta +\delta \right)⊉\bar{\Psi }\left(\alpha ,\;\beta \right)\cap \bar{\Psi }\left(\gamma ,\;\delta \right)$ . Since Im(Ψ) is a chain; implies that $\bar{\Psi }\left(\alpha +\gamma ,\;\beta +\delta \right)\subset \bar{\Psi }\left(\alpha ,\;\beta \right)\cap \bar{\Psi }\left(\gamma ,\;\delta \right)$ . Then, $\bar{\Psi }\left(\alpha +\gamma ,\;\beta +\delta \right)\in P\left([0,1]\right)$ . Clearly, [0,1]\Ψ(α+γ,β+δ)⊃([0,1]\Ψ(α,β))∩([0,1]\Ψ(γ,δ))=[0,1]\(Ψ(α,β)∪Ψ(γ,δ)) . So, Ψ(α+γ,β+δ)⊃Ψ(α,β)∪Ψ(γ,δ) . Take ε=Ψ(α+γ,β+δ) . Therefore, Ψ(α,β)⊂ε and Ψ(γ,δ)⊂ε . As a result, $\left(\alpha ,\;\beta \right),\;\left(\gamma ,\;\delta \right)\in F^{\subset }\left(\Psi ,\;\varepsilon \right)\neq \emptyset$ . Since $F^{\subset }\left(\Psi ,\;\varepsilon \right)$ is a congruence relation on Γ ; $\left(\alpha +\gamma ,\;\beta +\delta \right)\in F^{\subset }\left(\Psi ,\;\varepsilon \right)$ . So, Ψ(α+γ,β+δ)⊂ε=Ψ(α+γ,β+δ) . This is a contradiction. Thus, $\bar{\Psi }\left(\alpha +\gamma ,\;\beta +\delta \right)⊇\bar{\Psi }\left(\alpha ,\;\beta \right)\cap \bar{\Psi }\left(\gamma ,\;\delta \right)$ .
(ii) Let α,β,γ,δ∈Γ . To show that $\bar{\Psi }\left(\alpha ⋆\gamma ,\;\beta ⋆\delta \right)⊇\bar{\Psi }\left(\alpha ,\;\beta \right)\cap \bar{\Psi }\left(\gamma ,\;\delta \right)$ . Suppose that $\bar{\Psi }\left(\alpha ⋆\gamma ,\;\beta ⋆\delta \right)⊉\bar{\Psi }\left(\alpha ,\;\beta \right)\cap \bar{\Psi }\left(\gamma ,\;\delta \right)$ . Since Im(Ψ) is a chain; implies that $\bar{\Psi }\left(\alpha ⋆\gamma ,\;\beta ⋆\delta \right)\subset \bar{\Psi }\left(\alpha ,\;\beta \right)\cap \bar{\Psi }\left(\gamma ,\;\delta \right)$ . Then, $\bar{\Psi }\left(\alpha ⋆\gamma ,\;\beta ⋆\delta \right)\in P\left([0,1]\right)$ . Clearly, [0,1]\Ψ(α⋆γ,β⋆δ)⊃([0,1]\Ψ(α,β))∩([0,1]\Ψ(γ,δ))=[0,1]\(Ψ(α,β)∪Ψ(γ,δ)) . So, Ψ(α⋆γ,β⋆δ)⊃Ψ(α,β)∪Ψ(γ,δ) . Take ε=Ψ(α⋆γ,β⋆δ) . Therefore, Ψ(α,β)⊂ε and Ψ(γ,δ)⊂ε . As a result, $\left(\alpha ,\;\beta \right),\;\left(\gamma ,\;\delta \right)\in F^{\subset }\left(\Psi ,\;\varepsilon \right)\neq \emptyset$ . Since $F^{\subset }\left(\Psi ,\;\varepsilon \right)$ is a congruence relation on Γ ; $\left(\alpha ⋆\gamma ,\;\beta ⋆\delta \right)\in F^{\subset }\left(\Psi ,\;\varepsilon \right)$ . So, Ψ(α⋆γ,β⋆δ)⊂ε=Ψ(α⋆γ,β⋆δ) . This is a contradiction. Thus, $\bar{\Psi }\left(\alpha ⋆\gamma ,\;\beta ⋆\delta \right)⊇\bar{\Psi }\left(\alpha ,\;\beta \right)\cap \bar{\Psi }\left(\gamma ,\;\delta \right)$ .
(iii) Let α,β,γ,δ∈Γ and suppose that γ⋆δ≼α⋆β . To show that $\bar{\Psi }\left(\gamma ,\;\delta \right)⊇\bar{\Psi }\left(\alpha ,\;\beta \right)$ . Suppose that $\bar{\Psi }\left(\gamma ,\;\delta \right)⊉\bar{\Psi }\left(\alpha ,\;\beta \right)$ . Since Im(Ψ) is a chain; implies that $\bar{\Psi }\left(\gamma ,\;\delta \right)\subset \bar{\Psi }\left(\alpha ,\;\beta \right)$ . Then, [0,1]\Ψ(γ,δ)⊂[0,1]\Ψ(α,β) . Clearly, Ψ(γ,δ)⊃Ψ(α,β) . Take ε=Ψ(γ,δ) . Therefore, Ψ(α,β)⊂ε . As a result, $\left(\alpha ,\;\beta \right)\in F^{\subset }\left(\Psi ,\;\varepsilon \right)$ . Since $F^{\subset }\left(\Psi ,\;\varepsilon \right)$ is a congruence relation on Γ ; $\left(\gamma ,\;\delta \right)\in F^{\subset }\left(\Psi ,\;\varepsilon \right)$ . So, Ψ(γ,δ)⊂ε=Ψ(γ,δ) . This is a contradiction. Therefore, $\bar{\Psi }\left(\gamma ,\;\delta \right)⊇\bar{\Psi }\left(\alpha ,\;\beta \right)$ . Hence, $\bar{\Psi }$ is a hesitant fuzzy congruence relation on Γ .

## 6. Discussion

In this section, we explain the significance of hesitant fuzzy subalgebras, ideals, and congruences compared to traditional fuzzy sets or crisp sets. We also discuss practical situations or fields where these concepts are especially beneficial.

Hesitant fuzzy sets are valuable in any domain where there is uncertainty or hesitation in assigning precise membership values. Unlike traditional fuzzy sets and crisp sets, hesitant fuzzy sets provide a flexible framework for managing ambiguity in decision-making, pattern recognition, image processing, medical diagnosis, data mining, risk analysis, artificial intelligence, and control systems and robotics, among other applications. ^35^^–^^39^^,^^41^ Their ability to represent multiple membership values makes them particularly well-suited for complex, real-world problems where traditional fuzzy sets or crisp values may fall short.

Hesitant fuzzy subalgebras are useful for modeling systems where uncertainty affects internal consistency. For example, in cognitive processes or machine learning models, an object may partially belong to multiple substructures. Additionally, hesitant fuzzy ideals allow us to identify desirable or stable subsets within a system where boundaries are not sharply defined, which is especially useful in optimization and control. Finally, hesitant fuzzy congruences generalize equivalence relations for systems with vague class memberships, making them essential for tasks such as pattern recognition and fault-tolerant classification.

## 7. Conclusion

This paper introduced the study of hesitant fuzzy subalgebras of autometrized algebras. Also, we proved that a nonempty subset H of an autometrized algebra Γ is a subalgebra of Γ if and only if the characteristic hesitant fuzzy set $\chi_{H}$ is a hesitant fuzzy subalgebra of Γ . Further, we introduced the concept of the hesitant fuzzy ideal and examined some of its properties. We showed that hesitant fuzzy set χ on an autometrized algebra Γ is a hesitant fuzzy ideal of Γ if and only if for all ε∈P([0,1]) , a nonempty subset $F^{⊇}\left(\chi ,\;\varepsilon \right)$ is an ideal of Γ . Finally, we introduced a hesitant fuzzy congruence on autometrized algebras and discussed some of its properties. In particular, we explored a hesitant fuzzy equivalent relation $\bar{\Psi }$ on an autometrized algebra Γ is a hesitant fuzzy congruence relation on Γ if and only if for all ε∈P([0,1]) , $F^{\subseteq }\left(\Psi ,\;\varepsilon \right)$ is either empty or congruence relation on Γ .

In a future study, we will extend our research to interval-valued fuzzy subalgebras, ideals, and congruences, and investigate their algebraic characteristics. Interval-valued fuzzy sets enhance our ability to model and manage uncertainty and imprecision in complex systems. By incorporating a range of membership degrees, this approach enriches traditional fuzzy set theory, providing a more robust and flexible framework for decision-making in uncertain situations. This is especially relevant in fields such as risk assessment, medical diagnosis, environmental modeling, and engineering design, where precise data is often unavailable or incomplete. Despite theoretical richness, hesitant fuzzy sets are not widely adopted in industrial or real-world systems due to their complexity and the availability of simpler alternatives such as interval-valued fuzzy sets.

## Author contributions

All authors made equal contributions to this manuscript and have approved the final version.

## Ethics and consent

Ethics and consent were not required.

## Data Availability

There is no data associated with this article.
