# Supplementary material for: Hesitant Fuzzy Subalgebras, Ideals and Congruences on Autometrized Algebras
Source: F1000Res. 2025 Sep 9;14:183. Originally published 2025 Feb 10. [Version 2] doi: 10.12688/f1000research.161430.2 (PMC12441674; doi:10.12688/f1000research.161430.2)
Supplement: Peer review history

# Reviewer report: Reviewer response for version 2

**DOI:** 10.5256/f1000research.187101.r412595

**Published:** 2025-09-17

**Authors:** Ajoy Kanti Das

**Affiliations:** 1 Tripura University, Suryamani Nagar, Tripura, India

Thank you for sending me the revised version of the article *“Hesitant Fuzzy Subalgebras, Ideals and Congruences on Autometrized Algebras.” *I have carefully assessed the revisions, and the authors have addressed my earlier concerns. 

 Overall, the revisions significantly improve the clarity and quality of the paper. I find the manuscript suitable for approval.

Is the work clearly and accurately presented and does it cite the current literature?

Partly

If applicable, is the statistical analysis and its interpretation appropriate?

Not applicable

Are all the source data underlying the results available to ensure full reproducibility?

No source data required

Is the study design appropriate and is the work technically sound?

Yes

Are the conclusions drawn adequately supported by the results?

Yes

Are sufficient details of methods and analysis provided to allow replication by others?

Partly

Reviewer Expertise:

NA

I confirm that I have read this submission and believe that I have an appropriate level of expertise to confirm that it is of an acceptable scientific standard.

# Reviewer report: Reviewer response for version 2

**DOI:** 10.5256/f1000research.187101.r413664

**Published:** 2025-09-16

**Authors:** Jehad R Kider

**Affiliations:** 1 University of Technology-Iraq, Baghdad, Iraq

1.Generally, the paper is well written and has a good structure.

 2.The paper addresses a novel and underexplored intersection between hesitant fuzzy sets and autometrized algebra.

 3.The theoretical framework is built systematically, starting from definitions to illustrative examples and theorems.

 4.The work appears to be original and free of plagiarism, and no ethical concerns are noted

 Taking the above into consideration, I recommend the paper for indexing in your journal.

Is the work clearly and accurately presented and does it cite the current literature?

Yes

If applicable, is the statistical analysis and its interpretation appropriate?

Not applicable

Are all the source data underlying the results available to ensure full reproducibility?

No source data required

Is the study design appropriate and is the work technically sound?

Yes

Are the conclusions drawn adequately supported by the results?

Yes

Are sufficient details of methods and analysis provided to allow replication by others?

Partly

Reviewer Expertise:

fuzzy metric spaces, fuzzy normed spaces, fuzzy inner product spaces.

I confirm that I have read this submission and believe that I have an appropriate level of expertise to confirm that it is of an acceptable scientific standard.

# Reviewer report: Reviewer response for version 1

**DOI:** 10.5256/f1000research.177460.r391499

**Published:** 2025-06-20

**Authors:** Ajoy Kanti Das

**Affiliations:** 1 Tripura University, Suryamani Nagar, Tripura, India

**REVIEW REPORT**

**Title: Hesitant Fuzzy Subalgebras, Ideals and Congruences on Autometrized Algebras**

 This paper aims to extend the study of autometrized algebras by introducing the notions of hesitant fuzzy subalgebras, hesitant fuzzy ideals, and hesitant fuzzy congruences. It provides formal definitions, examples, and characterizations through theorems. The approach combines hesitant fuzzy set theory with abstract algebraic structures to fill gaps in the literature on fuzzy algebraic generalizations. The paper addresses a novel and underexplored intersection between hesitant fuzzy sets and autometrized algebra. The theoretical framework is built systematically, starting from definitions to illustrative examples and theorems. The inclusion of level sets and their algebraic properties adds depth to the analysis. The work appears to be original and free of plagiarism, and no ethical concerns are noted. However, there are several areas where the manuscript could benefit from improvement, both in terms of structure and content. Some comments are suggested to improve the quality of this paper.

**Suggestions for Improvement:**

1. The introduction lacks a clear motivation for the study, and no real-world relevance or potential applications are discussed. Include a deeper comparison with related works to position your model within the existing body of research. Emphasize the specific limitations in existing models that your work addresses.
2. Examples are presented without sufficient explanation, particularly in verifying algebraic closure in the operation tables.
3. Key definitions (e.g., hesitant fuzzy set operations, level set interpretation) are missing.
4. Some of the mathematical formulas use different styles or symbols that might be confusing to readers. It would be helpful to use consistent symbols throughout the paper and to explain each symbol clearly when it first appears. For example, symbols like “⊇”, “∩”, and “⊂” are used without explaining their meaning in the context of hesitant fuzzy sets. Often appears as “χ(α⋆β) ⊇ χ(α) ∩ χ(β)” without defining what the intersection of hesitant fuzzy sets means. Define all operations (intersection, union) explicitly in the hesitant fuzzy context.
5. The theoretical contributions in the paper are clear, but it would be helpful to explain why hesitant fuzzy subalgebras, ideals, and congruences are important or needed compared to traditional approaches. Including examples of real-world problems or areas where hesitant fuzzy set theory works better than classical methods would make the paper stronger. Consider adding a short section that discusses practical situations or fields where these concepts are especially useful. This would help justify the value of the theory.
6. The focus is primarily theoretical; real-world applications or potential use cases are not discussed. Expand the literature review by including more recent research on Fuzzy and Hesitant Fuzzy set theories and their applications in various domains. You may consider citing the following relevant and recent studies:

(refer to 1, 2, 3 4, 5 and 6) 

1. Include a future research section that outlines the next steps for further research to emphasize the study’s broader relevance and discuss any limitations of the current study.

The paper makes a significant theoretical contribution and has strong potential for real-world applications but requires minor revision before it can be accepted for indexing.

Is the work clearly and accurately presented and does it cite the current literature?

Partly

If applicable, is the statistical analysis and its interpretation appropriate?

Not applicable

Are all the source data underlying the results available to ensure full reproducibility?

No source data required

Is the study design appropriate and is the work technically sound?

Yes

Are the conclusions drawn adequately supported by the results?

Yes

Are sufficient details of methods and analysis provided to allow replication by others?

Partly

Reviewer Expertise:

Fuzzy set theory and its applications, Soft set and Decision-making, Soft Computing, Sequence space, Topology

I confirm that I have read this submission and believe that I have an appropriate level of expertise to confirm that it is of an acceptable scientific standard, however I have significant reservations, as outlined above.

## References

[1] Weighted hesitant bipolar-valued fuzzy soft set in decision-making. Songklanakarin Journal of Science and Technology, 45(6): 681-690 https://sjst.psu.ac.th/journal/45-6/10.pdf.

[2] An efficient water quality evaluation model using weighted hesitant fuzzy soft sets for water pollution rating. https://doi.org/10.1201/9781003494478-10.

[3] : An efficient water quality evaluation model using weighted hesitant fuzzy soft sets for water pollution rating.2025; 10.1201/9781003494478-10 179-195 10.1201/9781003494478-10

[4] : An Advanced Approach to Fuzzy Soft Group Decision-Making Using Weighted Average Ratings. *SN Computer Science* .2021;2(6) : 10.1007/s42979-021-00873-5 10.1007/s42979-021-00873-5

[5] A new fuzzy parameterized intuitionistic fuzzy soft multiset theory and group decision-making. Journal of Current Science and Technology, 12(3), 547-567. https://doi.org/10.14456/jcst.2022.42.

[6] IFP-intuitionistic multi fuzzy N-soft set and its induced IFP-hesitant N-soft set in decision-making, Journal of Ambient Intelligence and Humanized Computing, 14: 10143–10152. https://doi.org/10.1007/s12652-021-03677-w.

# Response

**Published:** 2025-08-22

**Authors:** Gebrie Yeshiwas Tilahun

**Affiliations:** Mathematics, Assosa University, Asosa, Benishangul-Gumuz, Ethiopia

We incorporated all the comments as the reviewer's suggestions. We add the following in the new version.

 1. In the introduction section, we are incorporating a clear motivation, real-world relevance and a deeper comparison of hesitant fuzzy set with related works.

 2. Examples are presented with sufficient explanation.

 3. The definitions of hesitant fuzzy set operations and level sets are discussed.

 4. We add a discussion section that explain the significance of hesitant fuzzy subalgebras, ideals, and congruences compared to traditional fuzzy sets or crisp sets. We also discuss practical situations or fields where these concepts are especially beneficial.

 5. The future scope of the research is also discussed.

 6. Recent reference materials suggested by you are incorporated.

# Reviewer report: Reviewer response for version 1

**DOI:** 10.5256/f1000research.177460.r366481

**Published:** 2025-03-11

**Authors:** Sileshe Gone Korma

**Affiliations:** 1 Arba Minch University, Hawassa, Ethiopia

This paper explores hesitant fuzzy subalgebras within autometrized algebras. It establishes that a nonempty subset of an autometrized algebra is a subalgebra if and only if its corresponding hesitant fuzzy set is a hesitant fuzzy subalgebra. The study also introduces the concept of hesitant fuzzy ideals and examines their properties. Additionally, it defines hesitant fuzzy congruences and investigates their characteristics, including conditions under which a hesitant fuzzy equivalence relation qualifies as a hesitant fuzzy congruence.

 The author should review the points I have highlighted in my comments and address them accordingly to improve clarity and accuracy.

1. Definition 2.3; condition (ii) is already satisfied in autometrized algebra Γ . Is there a possibility this condition fails for a subposet?
2. Definition 2.8, 2.9; what do you mean reference set?
3. Definition 2.3; it is better to write set complement *χ* *α* *=* *0,1* *-χ(α)*
4. Theorem 3.5 Proof(i) (b) and (c) it is better to combine both cases as *α∉H or β∉H* . The same is true for converse part (ii) (b) and (c)
5. Page 5 line 3 in the definition of *F* *⊇* *χ, ϵ* *={α∈*Γ *|* *χ* *(* *α* *)⊇* *ϵ* *}*  (in the brace both variables needs to be *α*  and check for the remaining)
6. If it is possible try to merge proof (ii) and (iii) as one for Theorem 3.6
7. Theorem 3.7 Proof(i) line 2 *χ(α+β)∈*Ρ[0,1]  is trivial please exclude it. The same is for  Proof(ii) line 2 .
8. Lemma 4.3 proof line 1 there is repetition please check it
9.  Lemma 4.3 line 2 typing error *χ* α*0 *=χ(α)*
10. Theorem 4.5 line 2 please add one line *χ(* α*β *0)⊇χ(α*0+β*0)  and remove *[* *0* *]*  in line 4
11. Theorem 4.6 Proof(i) (b) and (c) it is better to combine both cases as *α∉H or β∉H* . The same is true for converse part (ii) (b) and (c)
12. Definition 5.2 better to use as Ψ *a,b* *=*Ρ0,1 *-*Ψ *(* *a* *,* *b* *)*
13. Definition 5.2 Please write your definition as a hesitant fuzzy relation Ψ on Γ is said to be hesitant fuzzy equivalence relation if . . .  
14. Definition 5.6 line 1make it “ If Ψ is a  relation on Γ … “
15. Theorem 4.6 Proof(i) (b) and (c) it is better to combine both cases as *α∉H or β∉H* . The same is true for converse part (ii) (b) and (c)

Is the work clearly and accurately presented and does it cite the current literature?

Yes

If applicable, is the statistical analysis and its interpretation appropriate?

Not applicable

Are all the source data underlying the results available to ensure full reproducibility?

No source data required

Is the study design appropriate and is the work technically sound?

Yes

Are the conclusions drawn adequately supported by the results?

Yes

Are sufficient details of methods and analysis provided to allow replication by others?

Yes

Reviewer Expertise:

Algebra, lattice theory, universal algebra, fuzzy algebra

I confirm that I have read this submission and believe that I have an appropriate level of expertise to confirm that it is of an acceptable scientific standard.

# Response

**Published:** 2025-08-22

**Authors:** Gebrie Yeshiwas Tilahun

**Affiliations:** Mathematics, Assosa University, Asosa, Benishangul-Gumuz, Ethiopia

We have incorporated all the suggested comments and submitted the revised version. 

Answers for questions

 1. There is no possibility for the condition to fail as a subposet. This is simply an alternative definition of autometrized algebra intended to encompass all conditions. If we discard this definition, there is no difference.

 2. In this context, the term "reference set" simply refers to a universal set.
